# Antigen stimulation of peripheral blood mononuclear cells from *Mycobacterium bovis *infected cattle yields evidence for a novel gene expression program

**DOI:** 10.1186/1471-2164-9-447

**Published:** 2008-09-29

**Authors:** Kieran G Meade, Eamonn Gormley, Cliona O'Farrelly, Stephen D Park, Eamon Costello, Joseph Keane, Yingdong Zhao, David E MacHugh

**Affiliations:** 1 Comparative Immunology Group, School of Biochemistry and Immunology, Trinity College Dublin, Dublin 2, Ireland; 2 Tuberculosis Diagnostics and Immunology Research Centre, School of Agriculture, Food Science and Veterinary Medicine, College of Life Sciences, University College Dublin, Dublin 4, Ireland; 3 Animal Genomics Laboratory, School of Agriculture, Food Science and Veterinary Medicine, College of Life Sciences, University College Dublin, Dublin 4, Ireland; 4 Central Veterinary Research Laboratory, Backweston Campus, Celbridge, Co. Kildare, Ireland; 5 School of Medicine, Trinity College Dublin, St. James's Hospital, Dublin 8, Ireland; 6 Computational and Systems Biology Group, Biometric Research Branch, National Cancer Institute, Rockville, Maryland, USA; 7 Conway Institute of Biomolecular and Biomedical Research, University College Dublin, Dublin 4, Ireland

## Abstract

**Background:**

Bovine tuberculosis (BTB) caused by *Mycobacterium bovis *continues to cause substantial losses to global agriculture and has significant repercussions for human health. The advent of high throughput genomics has facilitated large scale gene expression analyses that present a novel opportunity for revealing the molecular mechanisms underlying mycobacterial infection. Using this approach, we have previously shown that innate immune genes in peripheral blood mononuclear cells (PBMC) from BTB-infected animals are repressed *in vivo *in the absence of exogenous antigen stimulation. In the present study, we hypothesized that the PBMC from BTB-infected cattle would display a distinct gene expression program resulting from exposure to *M. bovis*. A functional genomics approach was used to examine the immune response of BTB-infected (*n *= 6) and healthy control (*n *= 6) cattle to stimulation with bovine tuberculin (purified protein derivative – PPD-b) *in vitro*. PBMC were harvested before, and at 3 h and 12 h post *in vitro *stimulation with bovine tuberculin. Gene expression changes were catalogued within each group using a reference hybridization design and a targeted immunospecific cDNA microarray platform (BOTL-5) with 4,800 spot features representing 1,391 genes.

**Results:**

250 gene spot features were significantly differentially expressed in BTB-infected animals at 3 h post-stimulation contrasting with only 88 gene spot features in the non-infected control animals (*P *≤ 0.05). At 12 h post-stimulation, 56 and 80 gene spot features were differentially expressed in both groups respectively. The results provided evidence of a proinflammatory gene expression profile in PBMC from BTB-infected animals in response to antigen stimulation. Furthermore, a common panel of eighteen genes, including transcription factors were significantly expressed in opposite directions in both groups. Real-time quantitative reverse transcription PCR (qRT-PCR) demonstrated that many innate immune genes, including components of the TLR pathway and cytokines were differentially expressed in BTB-infected (*n *= 8) versus control animals (*n *= 8) after stimulation with bovine tuberculin.

**Conclusion:**

The PBMC from BTB-infected animals exhibit different transcriptional profiles compared with PBMC from healthy control animals in response to *M. bovis *antigen stimulation, providing evidence of a novel gene expression program due to *M. bovis *exposure.

## Background

*Mycobacterium bovis *infection is the cause of bovine tuberculosis (BTB), an important health problem in cattle that also has zoonotic potential for transmission to humans. The eradication of *M. bovis *infection in cattle is proving difficult in some developed countries, including the UK and Ireland [1] due to limitations in the sensitivity of current diagnostics, leading to a failure to detect all infected animals [2,3]. It is also unclear what role exposure to environmental mycobacterial antigens play in the generation of non-specific immune responses, giving rise to difficulties with test interpretation and reliability. Furthermore, some cattle with advanced disease become anergic, with suppressed cellular immune responses in both the peripheral blood and at the site of the infection [4], and remain undetected as reservoirs of disease.

The immune response to tuberculosis is a complex process and studies in the bovine model have primarily focused on the adaptive response. The outcome of tuberculosis infection depends on T cell interaction with macrophages [5], and progression of infection with *M. bovis *is thought to develop after a shift in the immune system from a protective proinflammatory, cytotoxic T cell response towards a non-protective antibody-mediated response [6,7]. The timing and potency of the cellular and immunological events that occur immediately post-infection are crucial determinants governing infection [6] and innate immune responses are considered important for the generation of early, and appropriate adaptive responses to resolve infection [8-11]. Therefore, central to the development of improved or novel diagnostics is increased understanding of the early immune response to tuberculosis in cattle.

At the most basic level, the interplay between the host and pathogen involves changes in gene expression [12]. High-throughput genomics technologies, which offer the ability to survey changes in expression for a large number of genes simultaneously, have been widely used to discern patterns of host gene regulation during infection. Microarray technology has emerged as the method of choice for large-scale gene expression studies that have increased our understanding of host-pathogen interactions [13-20,12]. These functional genomics studies have also identified new avenues of research for potential control strategies against pathogens [21]. Using this approach we have aimed to gain a better understanding of the molecular regulation of the immune response following *M. bovis *exposure and infection in cattle, with the expectation of significant benefits in development of new practical tools applicable to disease control [6].

We have previously used a bovine targeted immunospecific cDNA microarray to study gene expression changes in bovine peripheral blood mononuclear cells (PBMC) from BTB-infected cattle cultured *in vitro *in the presence of bovine and avian tuberculins [22]. PBMC are an accessible tissue for the development of robust novel diagnostics and previous studies have shown that for bovine tuberculosis, immune responses occurring in the peripheral blood reflect those at the site of disease [23]. In our previous study, antigen stimulation induced significant and specific expression changes in many immune genes that revealed different gene expression patterns in stimulated and non-stimulated PBMC. Although *IFNG *gene expression was increased in response to antigen stimulation, several other genes were highly expressed earlier in the time course [22], suggesting that these genes may represent valuable targets for the development of novel diagnostics of *M. bovis *in cattle early post-infection.

A subsequent comparative study between BTB-infected cattle and healthy control animal PBMC showed that the expression of innate immune genes is repressed in heavily infected cattle *in vivo*; it also demonstrated that the expression changes for many of these genes represent a BTB-signature of infection [24]. Additional expression profiling using real time qRT-PCR verified that a number of innate immune genes including *TLR2 *and *TLR4 *had reduced expression in BTB-infected animals [24]. Failure of *M. bovis *to activate these immune genes may have resulted from an antigen-induced suppression, or alternatively from a general failure of the immune system in the advanced diseased cattle.

For the present study, we compared PBMC gene expression profiles from natural *M. bovis*-infected animals with those from control non-infected animals after stimulation with bovine tuberculin (purified protein derivative – PPD-b) antigens. We hypothesized that the PBMC from BTB-infected cattle would display a distinct gene expression program resulting, at least in part, from previous exposure to *M. bovis*. These data provide evidence for a novel gene expression program in PBMC from infected animals and highlight the value of large-scale genomics approaches to understand immune regulatory mechanisms that in future may form the basis for novel diagnostics and therapeutics.

## Results

### Distinct microarray gene expression profile in PBMC from BTB-infected cattle at 3 h and 12 h post-stimulation with bovine tuberculin

**Figure 1:**
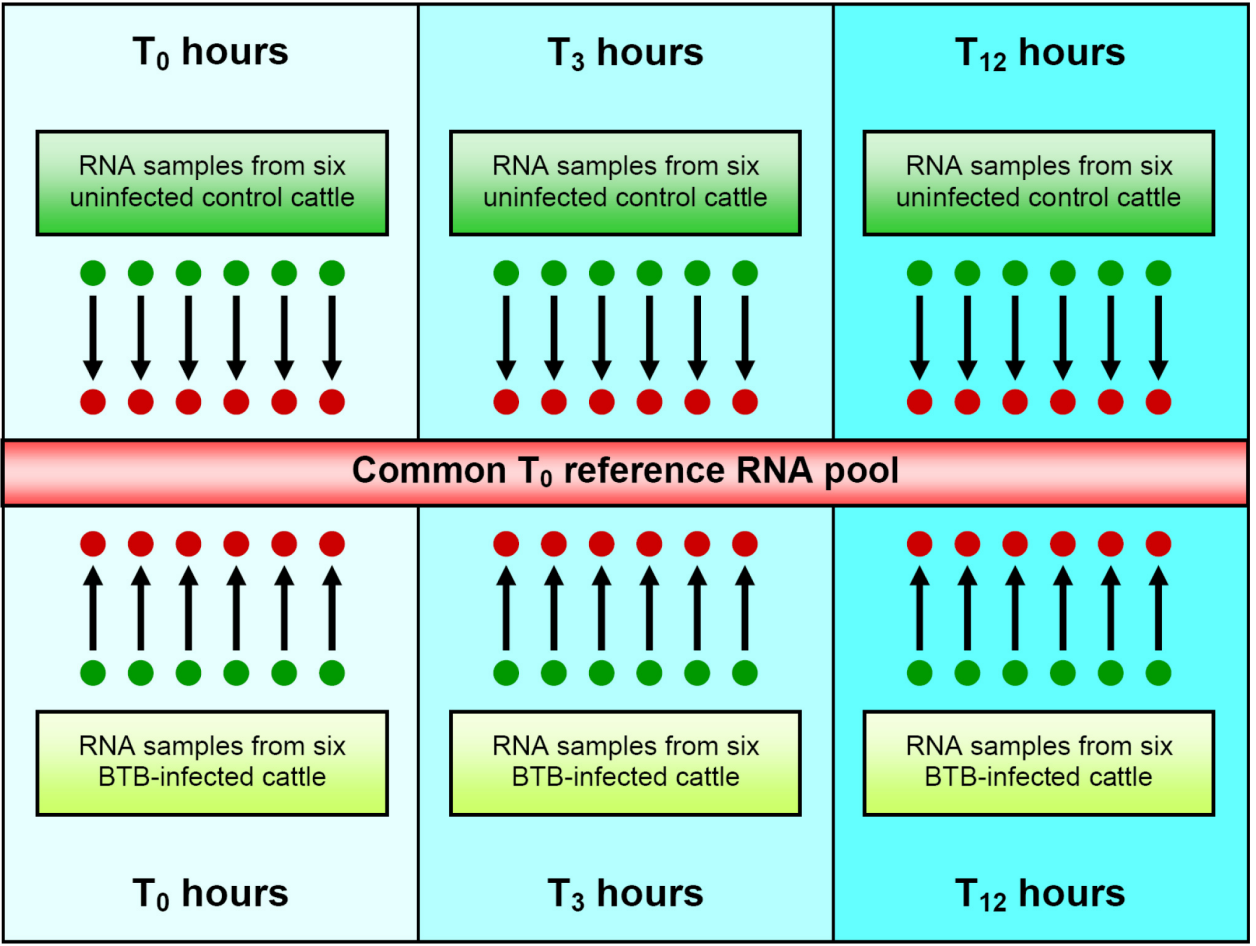
**Experimental design for BOTL-5 micro array hybridizations**. Common reference experimental design showing microarray hybridizations for six BTB-infected animals and six control non-infected animals across the 12-hour bovine tuberculin stimulation time course. In all cases, the test sample was labelled with Cy3 fluorescent dye (base of the arrow) and the common reference RNA (CRR) pool was labelled with Cy5 dye (arrow head). The CRR pool consisted of equal amounts of total RNA from the BTB-infected and control animal groups at T0 (0 h pre-stimulation) assembled as described previously [24].

Microarray analysis of mRNA expression levels was used to investigate the immune response differences between PBMC from *M. bovis *infected animals, compared to PBMC from control animals in response to stimulation with bovine tuberculin antigens (PPD-b). Gene expression profiling was performed on PBMC from six *M. bovis *infected cattle and six non-infected controls at three time points (pre-infection, and at 3 and 12 hours post-stimulation with bovine tuberculin), using a reference hybridization design and a common reference RNA (CRR) pool assembled as described previously [24] (Fig. 1). It is widely recognised that that a reference design is the most efficient in terms of resources and statistical flexibility for comparisons among different groups in multi-treatment experiments using dual colour microarrays [25-27]. The expression data generated was deposited in the NCBI Gene Expression Omnibus (GEO) repository [28] with experiment series accession [GEO:GSE12835]. Fold change values for all gene expression comparisons obtained from the microarray data were calculated as the mean of the two duplicate spots on the BOTL-5 microarray used for this study.

#### BTB-infected animals

**Figure 2:**
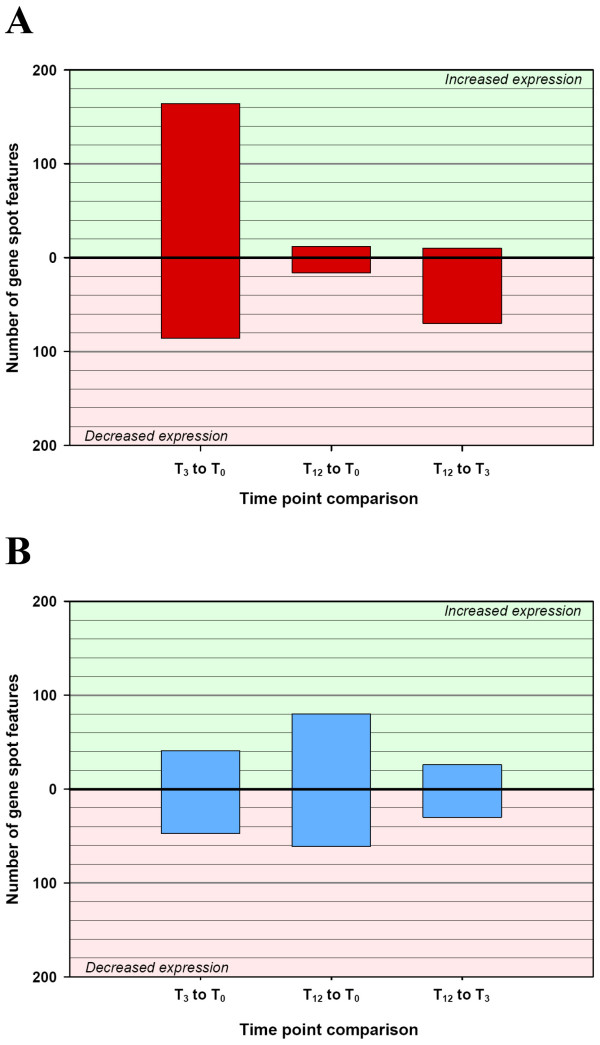
**Differentially expressed gene spot features between time points for the BTB-infected and control animal groups (*P *≤ 0.05)**. Significant differentially expressed gene spot features (*P *≤ 0.05) between time points (0 h, 3 h and 12 h) within the BTB-infected animal group (A) and the control non-infected animal group (B). The direction of the fold change, whether up or down, indicates a fold change difference between the first time point relative to the second time point.

Of the 1,391 duplicated genes (2,782 gene spot features) on the BOTL-5 microarray, 250 gene spot features (9.0%) showed significant differential expression in PBMC from BTB-infected animals after 3 h stimulation with bovine tuberculin, at the *P *≤ 0.05 level (see Additional file 1). A substantially smaller number of gene spot features (80) were differentially expressed in BTB-infected samples between 3 h and 12 h post-stimulation (Fig. 2a). Among the 250 differentially expressed spot features at 3 h post-stimulation, 164 were significantly increased in expression and 86 were decreased in expression relative to 0 h (*P *≤ 0.05 level). At 3 h post-stimulation with bovine tuberculin, the number of spot features showing increased expression outnumbered those with reduced expression by a ratio of 2:1. However, at 12 h post-stimulation the trend was reversed and there were seven-fold more spot features decreased in expression than increased, relative to 3 h post-stimulation (10 spot features showing increased expression versus 70 spot features showing decreased expression) [Fig. 2a; *P *≤ 0.05 level].

Inspection of these data revealed that the 250 early differentially expressed spot features included 83 unique genes where two replicate gene spot features were found to be significantly differentially expressed (6% of the 1,391 duplicated genes represented on the array). Fifty-three of these genes were identified as BOTL clones, ESTs derived from genes whose function(s) in cattle are unknown, but were inferred from evolutionary sequence homology with gene orthologs in rodents and humans (see Additional file 1). Similarly, between the later two time points, 18 genes were represented by significantly differentially expressed duplicate significant spot features (*P *≤ 0.05).

#### Control animals

In contrast to the differential gene expression profile observed in BTB-infected animals in response to bovine tuberculin stimulation, initial gene expression changes in the control PBMC samples were moderate with 88 spot features significantly differentially expressed between 0 h and 3 h (*P *≤ 0.05) [see Additional file 2]. These were almost evenly divided between increased (41) and decreased (47) expression at the later time point (Fig. 2b). Between 3 h and 12 h of stimulation, 56 spot features were differentially expressed (see Additional file 2), again almost equally balanced between repression and activation (26 spot features increased in expression and 30 decreased in expression) [Fig. 2b]. The initial 88 spot features and the later 56 spot features significantly differentially expressed represented only 14 and five unique genes represented by duplicate spot features (*P *≤ 0.05).

Fold changes for significantly differentially expressed genes on the microarrays ranged from a decrease of 4.38 fold (colony stimulating factor 2 receptor, alpha gene [*CSF2RA*] in the control animal group after 12 h stimulation with bovine tuberculin) to an increase in expression of 5.58 fold (the major histocompatability complex, class I, A gene [*BOLA*] in the BTB-infected animal group after 3 h stimulation with bovine tuberculin).

### Inversely correlated gene expression changes between BTB-infected and control PBMC after 3 h stimulation with bovine tuberculin

**Figure 3:**
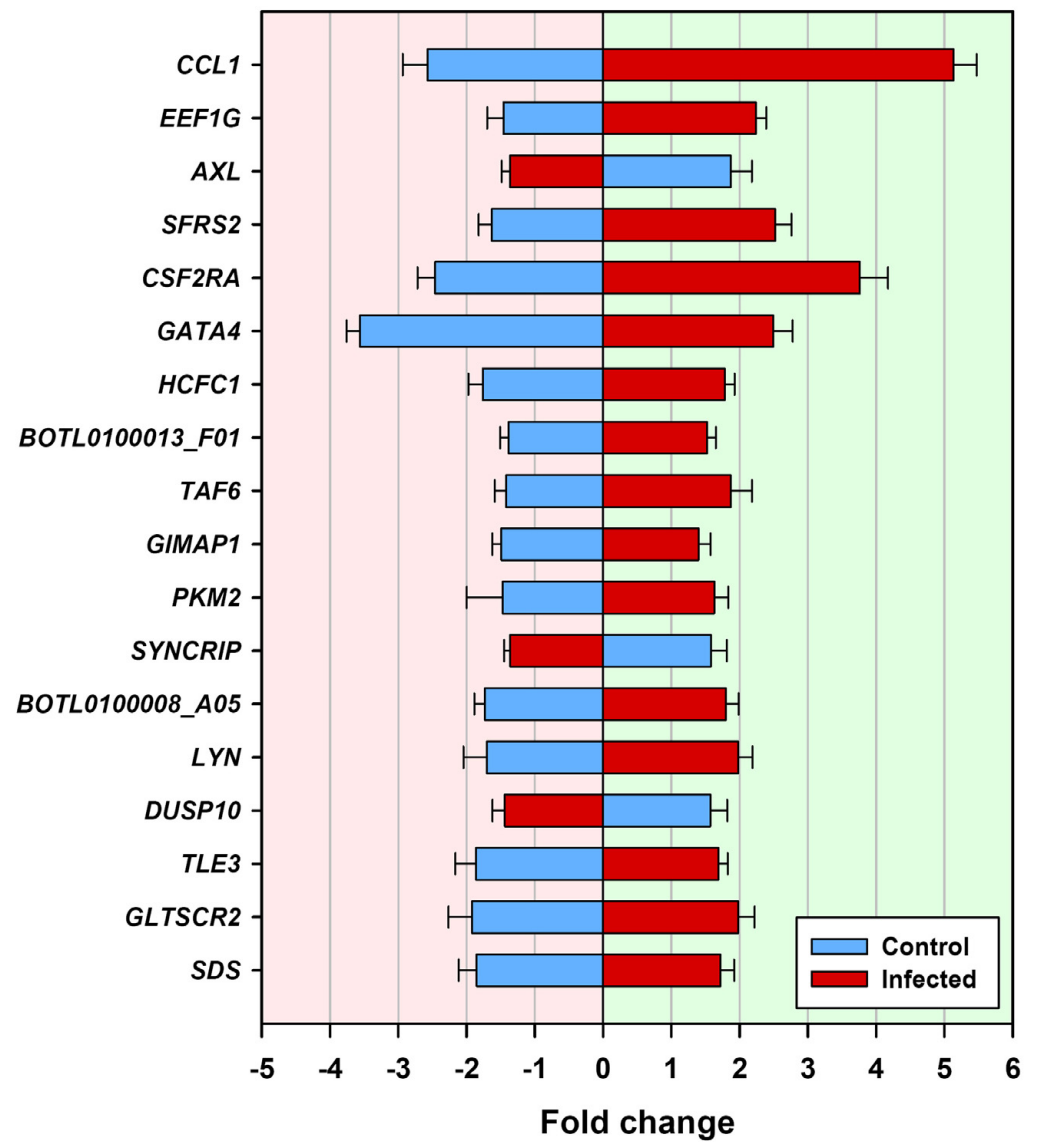
**Eighteen genes displaying a converse pattern of gene expression between control and BTB-infected animals [T_3 _relative to T_0_] (*P *≤ 0.05)**. Eighteen genes represented by duplicate significant microarray features (*P *≤ 0.05) that were differentially expressed between T_3 _(3 h post-bovine tuberculin stimulation) and T_0 _(0 h – no stimulation) for both the control and BTB-infected animal groups. Error bars show the standard error of the mean for each fold-change estimate.

Comparisons of the 250 significantly differentially expressed spot features in the BTB-infected animal group and 88 features from the control animal group identified 18 genes (represented by duplicate significant spot features) that were significantly differentially expressed in both groups in response to bovine tuberculin (*P *≤ 0.05, Fig. 3 and Table 1).

**Table 1:** Microarray gene expression fold change values for 18 significant genes within the non-infected control animal group (*n *= 6) and the BTB-infected animal group (*n *= 6) between T_0 _and T_3_.

**Gene symbol**	**Gene name**	**BOTL clone ID**	**Gene ontology (GO) function/s**	**Fold change infected cattle**	**Fold change control cattle**
*SDS*	Serine dehydratase	BOTL0100002XA02R	Amino acid metabolism	+1.72 ± 0.20	-1.85 ± 0.26
*GLTSCR2*	Glioma tumor suppressor candidate region gene 2	BOTL0100002XB07R	Unknown	+1.98 ± 0.24	-1.92 ± 0.34
*TLE3*	Transducin-like enhancer of split 3	BOTL0100003XG08R	Regulation of gene transcription	+1.69 ± 0.13	-1.86 ± 0.30
*DUSP10*	Dual specificity phosphatase 10	BOTL0100004XG06R	Phosphatase activity and response to stress	-1.44 ± 0.18	+1.57 ± 0.25
*LYN*	v-yes-1 Yamaguchi sarcoma viral related oncogene homolog	BOTL0100006XH06R	Amino acid phosphorylation and receptor signalling	+1.98 ± 0.21	-1.70 ± 0.34
*Unknown*	-	BOTL0100008_A05	Unknown	+1.80 ± 0.19	-1.73 ± 0.16
*SYNCRIP*	Synaptotagmin binding, cytoplasmic RNA interacting protein	BOTL0100009_E08	RNA binding	-1.36 ± 0.09	+1.58 ± 0.23
*PKM2*	Pyruvate kinase	BOTL0100010_C03	Protein binding and alternative splicing	+1.63 ± 0.21	-1.47 ± 0.52
*GIMAP1*	GTPase, IMAP family member 1	BOTL0100011_B07	Control of cell survival and nucleotide binding	+1.40 ± 0.18	-1.49 ± 0.13
*TAF6*	TAF6 RNA polymerase II, TATA box binding protein (TBP)-associated factor	BOTL0100012_G05	Regulation of transcription	+1.87 ± 0.31	-1.42 ± 0.17
*Unknown*	-	BOTL0100013_F01	Unknown	+1.52 ± 0.13	-1.38 ± 0.12
*HCFC1*	Similar to Host cell factor C1	BOTL0100013_G06	Regulation of transcription	+1.78 ± 0.15	-1.76 ± 0.21
*GATA4*	GATA binding protein 4	GATA binding protein 4 (GATA4)	Regulation of transcription	+2.49 ± 0.29	-3.56 ± 0.20
*CSF2RA*	Colony stimulating factor 2 receptor, alpha	colony stimulating factor 2 receptor, alpha)	Production, differentiation, and function of granulocytes and macrophages	+3.76 ± 0.41	-2.46 ± 0.26
*SFRS2*	Splicing factor, arginine/serine-rich 2	NBFGC_BE721857 chemokine (C-C motif) receptor 7 (CCR7)	mRNA processing	+2.52 ± 0.24	-1.63 ± 0.19
*AXL*	AXL receptor tyrosine kinase	NBFGC_BE722178 AXL receptor tyrosine kinase	Signal transduction	-1.36 ± 0.13	+1.87 ± 0.31
*EEF1G*	Eukaryotic translation elongation factor 1 gamma	NBFGC_BF230159 EEF1G eukaryotic translation elongation	Translational elongation	+2.24 ± 0.15	-1.46 ± 0.24
*CCL1*	Chemokine (C-C motif) ligand 1	Small inducible cytokine A1	Chemokine activity	+5.13 ± 0.34	-2.57 ± 0.36

Relative expression fold change values are shown with standard errors.

Clone IDs were obtained from the Center for Animal Functional Genomics (CAFG) website: .

The expression of 15 of these 18 genes was increased in the BTB-infected animals in response to bovine tuberculin stimulation relative to the unstimulated samples. All 18 genes were expressed in the opposite direction in the control animal group in response to bovine tuberculin stimulation, suggesting a common, but reversed mechanism affecting the regulation of expression or transcriptional trajectory of these genes in the two groups (Fig. 3 and Table 1). Among the 15 genes with significantly increased mRNA levels in PBMC from BTB-infected animals, and significantly decreased expression in PBMC from the control animals post-stimulation, were transcription factor genes (*TLE3*, *TAF6*, *HCFC1 *and *GATA4*) and genes coding for proteins involved in mRNA processing (*SFRS2*), nucleotide binding (*GIMAP1*), amino acid metabolism (*SDS*), amino acid phosphorylation (*LYN*), protein binding (*PKM2*), and translational elongation (*EEF1G*). Other genes with well-characterised roles in the immune response were also differentially expressed. These included the colony stimulating factor 2 receptor, alpha gene (*CSF2RA*), involved in the production, differentiation and function of myeloid cells, which was increased by 3.76 fold (*P *= 0.0057). The expression of the chemokine (C-C motif) ligand 1 gene (*CCL1*) was increased by 5.13 fold (*P *= 0.0009). The protein encoded by *CCL1 *is secreted by activated T cells and displays chemotactic activity for monocytes [29]. Three genes, an RNA binding gene (synaptotagmin binding, cytoplasmic RNA interacting protein–*SYNCRIP*), a phosphatase activity and stress response gene (dual specificity phosphatase 10–*DUSP10*) and a signal transduction gene (AXL receptor tyrosine kinase–*AXL*) were all reduced in their expression in PBMC from BTB-infected animals, but significantly increased in PBMC from control animals in response to bovine tuberculin stimulation (Table 1).

### Rapid but transient proinflammatory response to bovine tuberculin in PBMC from BTB-infected animals detected at 3 h post stimulation

Of the 1,391 genes on the BOTL-5 microarray, 250 spot features showed significant differential expression between the BTB-infected animals at 0 h and 3 h post stimulation at the *P *≤ 0.05 level (see Additional file 1). Conversely, only 80 spot features were differentially expressed between the later two time points (Fig. 2a). It is clear from the number of genes differentially expressed in PBMC from BTB-infected samples that the majority of differential gene expression occurred early, within 3 h of stimulation with tuberculin antigens. This response was both immediate and transient (Fig. 2a). In contrast, the magnitude of gene expression changes in control animals was significantly different than in the PBMC from BTB-infected samples in response to bovine tuberculin stimulation. Furthermore, the profile of expression indicated that the majority of differential expression occurred between 0 h and 12 h in PBMC from the control animals (Fig. 2b).

A number of genes with well characterised roles in infection and immunity were found to be significantly differentially expressed in the BTB-infected animal group after stimulation with bovine tuberculin. Some of those that were significantly increased in expression by 3 h were the fibroblast growth factor receptor genes (*FGFR1*) and (*FGFR2*) [2.27 and 1.91 fold respectively], the colony stimulating factor 2 receptor, alpha gene (*CSF2RA*) [3.76 fold], the lymphotoxin alpha gene (*LTA*) [1.76 fold], and a number of kinase genes: *AKT1 *(2.38 fold), *MAP2K1 *(1.59 fold), *MAP2K7 *(2.58 fold), *MAPKAPK2 *(1.71 fold), *MAP4K2 *(1.62 fold), *MLK3 *(3.80 fold), *PIK3R5 *(1.53 fold) and *KSR1 *(2.44 fold). The results from the microarray data also highlighted the involvement of a number of genes encoding components of the TLR signalling pathway including *CD14 *(2.48 fold), *IKBKB *(1.98 fold) and *NFKB1 *(2.32 fold), all of which were significantly increased in expression (*P *≤ 0.05). A significant increase in a gene involved in antigen presentation (BOLA major histocompatibility complex-class I, A–*BOLA-A*) was also detected (5.43 fold, *P *≤ 0.05).

Among those differentially expressed genes that were expressed at lower levels at 3 h post-stimulation with bovine tuberculin were a number of BOTL clones with homology to known genes. These included *PPIA *(-1.68 fold) and the protein phosphatase genes, *PPP2CA *(-1.44 fold), *PPP6C *(-1.41 fold) and *PTPRF *(-1.26 fold).

In contrast, 88 gene spot features were significantly differentially expressed in response to bovine tuberculin stimulation in the control animals, 41 of these with increased expression levels and 47 expressed at lower levels (*P *≤ 0.05, Fig. 2b). Only 14 genes within this group were represented by duplicate spot features. Among the genes that were significantly increased in expression in response to bovine tuberculin stimulation were *AXL *(1.87 fold) and *MAPK13 *(1.84 fold). Genes with significantly decreased expression in response to stimulation were *GATA4 *(-1.83 fold), *MIF *(-1.40 fold), and *CCR7 *(-1.63 fold).

### Real-time quantitative reverse transcription PCR (qRT-PCR) supports a differential role for TLR-associated molecules in the early response of BTB-infected animals to bovine tuberculin

An extended panel of 48 PBMC mRNA samples (representing eight infected and eight non-infected control animals, unstimulated and at 3 h and 12 h post-stimulation with bovine tuberculin) were used for real time qRT-PCR validation studies. The differentially expressed genes that were detected were classified using gene ontology (GO). Selected genes supplemented with others from relevant literature in human and murine models of TB, including those encoding molecules involved in pathogen recognition (*TLR2, TLR4 *and *IL1R*), signal transduction (*MYD88, TOLLIP*, and *TICAM2*), gene transcription (*NFKB1*) and cytokine production (*IFNG, IL8 *and *IL10*) were all investigated by real time qRT-PCR. The results from the 41 genes used for these single gene expression studies are detailed in Tables 2 and 3 and corroborate the results obtained from the BOTL-5 hybridisations.

**Table 2:** Gene expression fold change differences for the BTB-infected animal group (*n *= 8) between T_3 _and T_0 _validated using real time qRT-PCR.

**Gene symbol**	**Gene name**	**Gene ontology (GO) function/s**	**Fold change difference**	***P*-value**
*IFNG*	Interferon γ	Cytokine activity	+16.62 ± 8.74	0.0012
*AKT1*	V-akt murine thymoma viral oncogene homolog 1	Protein kinase activity	+4.12 ± 0.46	0.0000
*IKBKB*	Inhibitor of kappa light polypeptide gene enhancer in B-cells, kinase beta	Transcription activator activity	+3.64 ± 0.61	0.0000
*NCOR1*	Nuclear receptor co-repressor 1	Transcriptional repression	+2.80 ± 0.46	0.0001
*IL1RN*	Interleukin 1 receptor antagonist	Signal transduction	+2.79 ± 0.60	0.0012
*TOLLIP*	Toll interacting protein	Signal transduction	+2.33 ± 0.34	0.0002
*LTA*	Lymphotoxin alpha (TNF superfamily, member 1)	Cytokine activity	+2.27 ± 0.75	0.0135
*TLR4*	Toll-like receptor 4	Bacterial binding and signal transduction	+2.24 ± 0.36	0.0037
*MAPKAPK2*	Mitogen-activated protein kinase-activated protein kinase 2	Signal transduction	+2.12 ± 0.28	0.0001
*IL8*	Interleukin 8	Cytokine activity	+2.09 ± 0.19	0.0000
*IL1A*	Interleukin 1α	Cytokine activity	+1.73 ± 0.22	0.0021
*NFKB1*	Nuclear factor κβ	Transcriptional activation	+1.40 ± 0.42	0.0082
*CHUK*	Conserved helix-loop-helix ubiquitous kinase	Signal transduction	+1.38 ± 0.09	0.0009
*TICAM2*	Toll-like receptor adaptor molecule 2	Signal transduction	+1.28 ± 0.07	0.0006
*IL10*	Interleukin 10	Cytokine activity	+1.24 ± 0.09	0.0095
*TGFB1*	Transforming growth factor, beta 1	Protein binding	+1.19 ± 0.34	0.0021
*TNF*	Tumor necrosis factor (TNF superfamily, member 2)	Cytokine activity	+0.94 ± 0.48	0.0314
*IL1R2*	Interleukin 1 receptor 2	Cytokine activity	-1.56 ± 0.63	0.0012

Relative expression fold change values are shown with standard errors.

**Table 3:** Gene expression fold change differences for the control non-infected animal group (*n *= 8) between T_3 _and T_0 _validated using real time qRT-PCR.

**Gene symbol**	**Gene name**	**Gene ontology (GO) function/s**	**Fold change difference**	***P*-value**
*TOLLIP*	Toll interacting protein	Signal transduction	+4.32 ± 1.87	0.0094
*IRAK1*	Interleukin-1 receptor-associated kinase 1	Transcription activator activity	+3.89 ± 1.91	0.0492
*IL10*	Interleukin 10	Cytokine activity	+2.00 ± 0.69	0.0091
*IL1R1*	Interleukin 1 receptor, type 1	Protein binding and signal transduction	+1.61 ± 0.55	0.0182
*IL1RN*	Interleukin 1 receptor antagonist	Protein binding and signal transduction	+1.53 ± 0.62	0.0067
*TLR2*	Toll-like receptor 2	Bacterial binding and signal transduction	+1.37 ± 0.06	0.0000
*MYD88*	Myeloid differentiation primary response gene (88)	Signal transduction	+1.23 ± 0.78	0.0489
*IL1A*	Interleukin 1α	Cytokine activity	+0.83 ± 0.45	0.0367

Relative expression fold change values are shown with standard errors.

**Figure 4:**
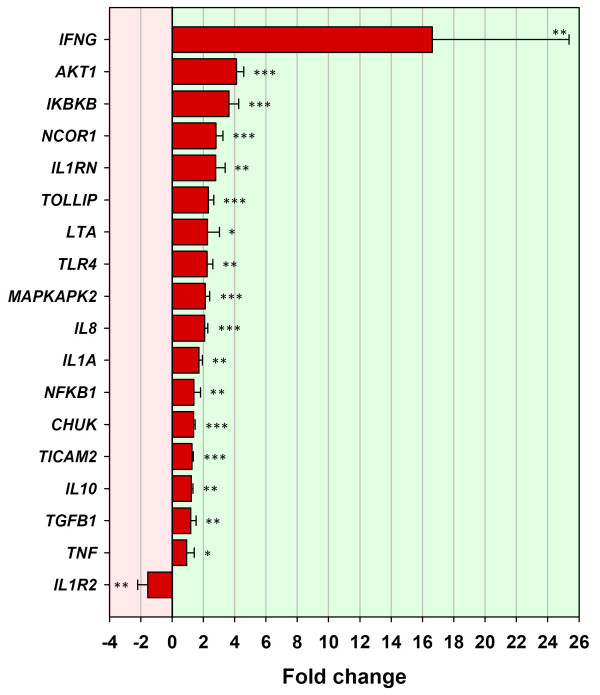
**Genes validated using real time qRT-PCR for the BTB-infected animal group (T_3 _relative to T_0_)**. Eighteen genes validated using real time qRT-PCR for the BTB-infected animal group between T_3 _(3 h post-bovine tuberculin stimulation) and T_0 _(0 h – no stimulation). Error bars show the standard error of the mean for each fold change estimate. Statistical significance for each gene is shown as follows: *P ≤ 0.05; **P ≤ 0.01; ***P ≤ 0.001.

Expression levels of *CD14 *(from the BOTL-5 microarray data) and *TLR4 *in the BTB-infected animal group were increased by 2.48 and 2.24 fold respectively (*P *= 0.0333 and *P *= 0.0037). Genes encoding downstream components of the TLR pathway were also examined by real time qRT-PCR, and increased mRNA abundance for *CHUK *and *IKBKB*, further verified the array data (1.38 and 3.64 fold respectively; *P *= 0.0009 and *P *= 0.0000). This also suggested a mechanism to explain the increased expression of *NFKB1 *by 1.4 fold (*P *= 0.0082). A significant 16.62 fold increase in the mRNA expression of the proinflammatory cytokine gene, *IFNG *(*P *= 0.0012) was also detected (Fig. 4 and Table 2).

**Figure 5:**
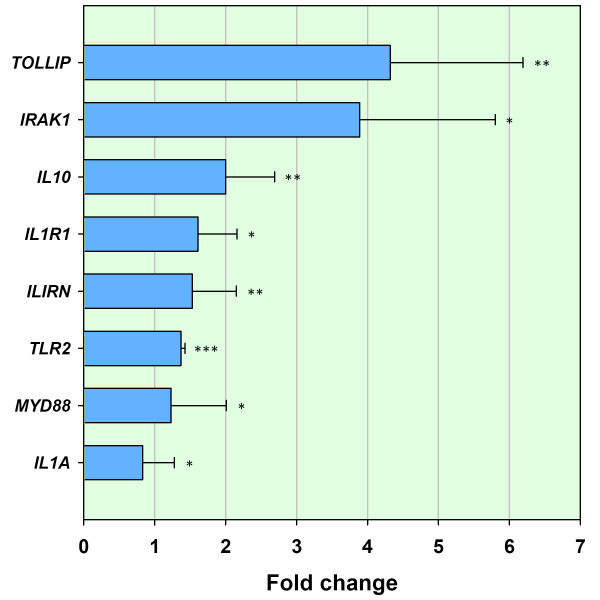
**Genes validated using real time qRT-PCR for the control animal group (T_3 _relative to T_0_)**. Eight genes validated using real time qRT-PCR for the control animal group between T_3 _(3 h post-bovine tuberculin stimulation) and T_0 _(0 h – no stimulation). Error bars show the standard error of the mean for each fold change estimate. Statistical significance for each gene is shown as follows: *P ≤ 0.05; **P ≤ 0.01; ***P ≤ 0.001.

In contrast, for the control animal group, increased expression of *TLR2 *by 1.37 fold (*P *= 0.0000) and *MYD88 *by 1.23 fold (*P *= 0.0489) was noted (Fig. 5 and Table 3). In addition, *TLR2 *and *MYD88 *were examined in the infected group by real time qRT-PCR and *TLR4 *in the control group; all three genes were found not to be differentially expressed (data not shown). Genes involved in the interleukin 1α receptor signalling pathway were also differentially expressed in both treatment groups. Increased expression of the interleukin 1α gene (*IL1A*) and the interleukin 1 receptor type I gene (*ILIRI*) was detected in the control animal samples by 0.83 and 1.61 fold respectively (*P *= 0.0367 and *P *= 0.0182). The IL-1R antagonist gene (*IL1RN*) was also increased in expression by 1.53 fold (*P *= 0.0067). While *IL1A *and *IL1RN *were also significantly increased in the BTB-infected animal samples (1.73 and 2.79 fold respectively; *P *= 0.0021 and *P *= 0.0012), the decoy receptor *IL1R2 *was differentially expressed by -1.56 fold (*P *= 0.0000, Fig. 5). Expression of the IL-1R1 gene (*IL1R1*) was not significantly increased (data not shown).

Toll interacting protein (encoded by *TOLLIP*) is a negative regulator of the TLR pathway and has been characterised in humans and mice [30]. When examined by real time qRT-PCR, it was found to be significantly increased in expression in PBMC of both BTB-infected animal (Fig. 4) and the control samples (Fig. 5) after bovine tuberculin stimulation by 4.32 and 2.33 fold respectively (*P *= 0.0094 and *P *= 0.0002). Expression levels of the gene encoding a TLR4-specific molecule, Toll-like receptor adaptor molecule 2 (*TICAM2*) [31], were estimated in both groups and although not differentially expressed in the control animal group (data not shown), it was significantly increased by 1.28 fold in the BTB-infected animal group (*P *= 0.0006, Fig. 4).

The gene encoding interleukin-10 (*IL10*) was significantly differentially expressed in both groups (Figs. 4 and 5). In the BTB-infected animal group, *IL10 *expression was increased by 1.24 fold (*P *= 0.0135). However, in the control animal group, *IL10 *was significantly increased in expression by 2.00 fold (*P *= 0.0091). In addition, it was noted that neither *NFKB *nor *IFNG *were differentially expressed in the control animal group when tested by real time qRT-PCR (data not shown).

## Discussion

Bovine tuberculosis is the fourth most important livestock disease worldwide [32]. The benefits of developing, applying and maintaining improved control and eradication strategies for BTB are manifold, and directly impact on human and animal health [33]. The specific immune cell signalling pathways involved in the immune response to BTB are highly complex and poorly characterised in cattle. This has obvious limitations for the understanding of, and design of improved diagnostics and effective therapeutics. However, studies on tuberculosis in the human and murine models have highlighted the involvement of cell regulatory signalling pathways in the immune response that are also likely to be relevant in BTB. Whereas traditionally, studies of BTB have focused on adaptive immunity, the findings from these studies are pointing toward a critical role for signalling via the innate immune system, including TLRs in initiating and directing the subsequent immune response and determining the outcome of infection [8-10].

The timing and potency of the cellular and immunological events that occur immediately post-infection are crucial determinants governing infection [6]. Pathogen-induced phenotypic changes in host cells are often accompanied by marked changes in gene expression due to host- and/or pathogen mediated reprogramming of the transcriptome during infection [34]. Previous work by our group compared the gene expression differences between bovine tuberculin-stimulated and non-stimulated PBMC from BTB-infected animals [22] and demonstrated that stimulation with bovine tuberculin induced significant gene expression changes that can be useful for dissection of the immune response to BTB. Subsequently we identified a novel gene expression profile indicative of innate immune gene repression in heavily infected cattle *in vivo *[24]. Expression clustering of these data yielded a gene infection signature for disease and highlighted genes and regulatory pathways, including the TLR cell signalling pathway [24].

In the present study we have shown that after overnight recovery *in vitro*, PBMC from BTB-infected cattle are significantly more responsive to bovine tuberculin stimulation than control animal PBMC. Gene expression levels were estimated in PBMC from BTB-infected and healthy controls either non-stimulated or at 3 h and 12 h after bovine tuberculin stimulation using a common reference microarray approach (Fig. 1). Significant gene expression changes were observed in response to bovine tuberculin stimulation for both animal groups over a 12 h time course. The microarray data indicated that substantially different gene expression profiles were evident in the BTB-infected animals relative to the control animals (Fig. 2a and Fig. 2b). Following a 12 h incubation with bovine tuberculin, these analyses also showed that the immune response in the infected animal group was both rapid and transient (Fig. 2a). Analysis of gene expression differences across the time course showed that differences between groups were most evident in the period between 0 h and 3 h after bovine tuberculin stimulation–indicating that the early response to bovine tuberculin is substantially different between the two animal groups.

Comparative analysis of the PBMC gene expression program in response to bovine tuberculin identified a panel of 18 genes that were significantly differentially expressed in both animal groups. Interestingly, all of these genes were expressed in opposite directions in the two groups. Expression of 15 of the 18 genes was increased in PBMC from BTB-infected animals including genes encoding proteins involved with transcriptional regulation (*TLE3, TAF6, HCFC1 *and *GATA4*), a chemokine (*CCL1*), and a chemokine receptor(*CSF2RA*). In contrast, only three genes were decreased in expression in the BTB-infected group. Conversely, the opposite pattern was observed in PBMC from the control animals with 15 genes decreased, and three genes increased in expression relative to the BTB-infected group (Fig. 3 and Table 1). These data suggest a number of important gene targets for further study, as well as identifying cell regulatory pathways that may be differentially regulated in BTB-infected animals.

Of the 250 microarray spot features that were differentially expressed in the BTB-infected animals, those displaying increased expression in response to bovine tuberculin outnumbered those with decreased expression by a factor of two. We have previously shown that the expression of a number of the genes represented by these spot features, including *TLR2*, *TLR4 *and *NFKB*, was significantly repressed in heavily infected cattle *in vivo *[24]. The transformation associated with gene repression *in vivo *to gene activation detected in the same animals *in vitro *was evident in the reversal in direction of expression of a number of well characterised genes including those encoding TLRs, MHC molecules, and cytokines. Furthermore, the 41 genes examined by real time qRT-PCR confirmed the BOTL-5 microarray results and supported a trend towards a proinflammatory immune response to bovine tuberculin in PBMC from BTB-infected cattle. Expression of a gene encoding the key transcription factor and mediator of the immune response (*NFKB1*) indicated a distinctive proinflammatory gene expression program characterised by a significant two-fold increase in *IL8 *expression and 16-fold increase in *IFNG *expression (Fig. 4 and Table 2).

While the differentially expressed genes detected in this study provide evidence of a pre-existing gene expression program most likely caused as a result of *M. bovis *infection in the diseased cattle, other confounding factors could be responsible for some of the changes detected. Changes or fundamental differences in cell subpopulations between animals and the separation of PBMC from an *in vivo *immunosuppressive environment [24] into culture media may have affected some gene expression patterns. Although there was no evidence at post-mortem examination of clinical disease in the cattle caused by other infectious agents, the presence of undetected pathogens may have generated host immune responses in either group of cattle that could have accounted for some of the transcriptional changes detected. In addition, it is important to acknowledge that the control and infected cattle were sampled from different herds, and that this may represent a confounding factor in the analysis of gene expression differences between the groups.

Genes encoding adaptor and mediator molecules of the TLR activation pathway were also profiled by real time qRT-PCR to examine cellular pathways contributing to the differential response between BTB-infected and control animal groups. The gene encoding the Toll-interacting protein (TOLLIP), a negative regulator of TLR signalling [30] was examined and gene expression was significantly increased in both the control and BTB-infected animal groups (4.32 fold and 2.33 fold, respectively) in response to bovine tuberculin stimulation. TOLLIP has previously been found to impair TLR-2 and TLR-4 activation of NF-κB [30]; therefore, it might be expected to prevent the downstream signalling from TLR-2 and TLR-4 in healthy control and BTB-infected cattle samples respectively. However, significant increased expression of *NFKB1 *and genes encoding mediators of NF-κB (*CHUK *and *IKBKB*, Fig. 4 and Table 2), coupled with differential expression of over 250 spot features on the microarrays suggested that proinflammatory signalling was not inhibited in the BTB-infected animal samples. Interestingly, another TLR-4-specific accessory molecule, Toll-like receptor adaptor molecule 2 (TICAM2)[31], discovered in studies of *M. tuberculosis *infection in mice can bypass the inhibitory effects of TOLLIP to transmit cell signals [35] leading to increased expression of the *NFKB1 *gene [31]. Expression of the *TICAM2 *gene was examined using real time qRT-PCR and was found to be increased in the BTB-infected animal samples only (1.28 fold, Fig. 4 and Table 2). The increased expression of *TICAM2 *may possibly provide a mechanism through which proinflammatory gene activation is regulated in BTB-infected animals (Fig. 2a).

Our results are consistent with proposed mechanisms that drive an ineffective T_H_2 type response and contribute to the outcome of BTB infection in cattle. It has been suggested that the T_H_1/T_H_2 bias of the immune response can be determined by specific TLRs [36]. Furthermore, TLR-2 activation is a less efficient method of proinflammatory gene activation and may play a role in TLR-2 mediated immunosuppression [37].

In the present study, increased expression of the *IL10 *and *TGFB1 *genes was detected in the BTB-infected group in response to bovine tuberculin stimulation (Fig. 4) and elsewhere this has been associated with decreased ability of PBMC and macrophages to restrict mycobacterial growth in both humans [38,39] and mice [40,41]. Furthermore, IL-10 has been implicated in the suppression of the proinflammatory immune response and subversion of the host bacteriocidal immune response [42]. The results of the present study suggest that the 16-fold increased expression of the *IFNG *gene (Table 2) may drive the changes in gene expression detected in the BTB-infected animal group. In addition, although the *IL10 *gene is significantly increased in expression by 1.23 fold (Table 2), this may be insufficient to compete with the proinflammatory effects of IFN-γ. In this regard, it has been previously shown that the ratio of pro- and anti-inflammatory cytokines will determine the overall outcome of the immune response and subsequent correlated gene activation and/or repression [43,11].

In many countries the presence of *M. bovis*-infected wildlife can act as reservoirs of BTB infection for livestock and there is increased information available on the host response to infection in a variety of these species [44,45]. In our previous study [24], transcriptional profiling of infected and non-infected control animals in the absence of exogenous antigen stimulation demonstrated decreased expression of MHC class II molecules, and similar findings have been reported in deer in response to natural TB infection [46].Thacker and colleagues characterised the immunological responses of peripheral blood leukocytes (PBL) from *M. bovis*-infected and non-infected white-tailed deer to infection by monitoring cytokine gene expression after exogenous antigen stimulation [44]. The infected deer displayed a significant 75-fold increase in the expression of the proinflammatory cytokine, IFN-γ, comparable to the response detected in cattle during the present study. One notable difference was that the increase in *IL10 *and *TGFB1 *gene expression in response to bovine tuberculin stimulation of PBMC from infected cattle was not detected in infected deer. However, post-stimulation time points differed between the two studies and this could account for the differences observed.

The results of the present study are consistent with work carried out on Johne's disease in cattle caused by *M. avium *subsp. *paratuberculosis *(MAP) [47,48]. Those studies also detected a novel gene expression profile in PBMC from MAP-infected animals that was both rapid and transient across time [47,48]. In a separate study, stimulation of PBMC with MAP was shown to suppress the proinflammatory immune response [49], and the authors also found evidence of a dynamic T_H_1/T_H_2 type response, which eventually gives way to a predominantly T_H_2 like response in MAP-infected animals [50]. Such a temporal expression pattern, where peak production of IL-10 lags behind that of IFN-γ, has also been demonstrated in relation to MAP stimulation of bovine PBMC [50]. An important outcome from these studies involving both MAP-infected and BTB-infected cattle, is that the host gene expression profiles resulting from infection by these two related mycobacteria are remarkably similar.

## Conclusion

The outcome of infection with *M. bovis *is determined by a complex and dynamic interaction between the host immune system and the pathogen [51]. The cellular signalling events governing the immune responses that form the basis of cell-mediated immune BTB diagnostic tests remain incompletely understood. We hypothesized that the PBMC from BTB-infected cattle would display a distinct gene expression program resulting, from previous exposure to *M. bovis*. The gene expression program observed in PBMC from BTB-infected cattle was substantially different than in PBMC from control animals suggesting that BTB infection can modulate the immune response to bovine tuberculin. To the best of our knowledge, this work is also among the first to report the involvement of TLRs and TLR accessory molecules in this immune response.

The results demonstrate that PBMC from BTB-infected animals are highly responsive to bovine tuberculin stimulation *in vitro*, due possibly to their removal from an immunosuppressive environment *in vivo *[24]. Specific genes are commonly involved in the differential response of both control and BTB-infected animals indicating coordinated regulation of the innate immune response to antigen stimulation. The differential responses of a number of genes involved in cell signalling pathways regulating both the innate and adaptive immune response is indicative of a pre-existing gene expression program. The results are in agreement with other human and mouse models using *M. tuberculosis *[52-55] and bovine studies using MAP. The specific genes activated in response to mycobacterial infection have yet to be fully elucidated, but the overall response profiles seem to be similar among different mammalian hosts (*Homo sapiens, Mus musculus *and *Bos taurus*) infected with mycobacterial pathogens (*M. tuberculosis*, *M. bovis *and *M. avium *subsp. *paratuberculosis*). The results also demonstrate the usefulness of employing the natural host for *M. bovis *infection as a model to investigate the immune response to tuberculosis using functional genomics technologies.

Future work will concentrate on the elucidation of cell signalling pathways detected during the immune response to bovine tuberculin and their potential roles in the immune repression detected *in vivo *[24]. It is clear that a pre-existing immune program in PBMC from BTB-infected animals influences the response to bovine tuberculin stimulation and may affect the outcome of infection. Understanding the causes and consequences of this novel gene expression program should ultimately lead to a more complete understanding of the immune response to BTB and inform the development of novel or improved future diagnostics and vaccines.

## Methods

### Experimental animals and infection status

Sixteen cattle were used for this study. The eight infected animals were chosen from herds with a history of *M. bovis *infection. The animals were selected on the basis of the skin-fold thickness response to bovine and avian tuberculin in the single intradermal comparative tuberculin test (SICTT). The SICTT reactor animals were selected where the skin-fold thickness response to PPD-bovine exceeded that of PPD-avian by at least 12 mm. All of these animals were also measured positive in a whole blood IFN-γ assay [56]. The cattle were confirmed positive for tuberculosis following detailed *post-mortem *pathological examination and/or culture. There was no evidence at post-mortem examination that any of the BTB-infected animals had clinical diseases caused by other infectious agents. Bronchial, mediastinal, submandibular, retropharyngeal, mesenteric and hepatic lymph nodes and lungs were examined macroscopically for tuberculosis lesions. Suspected lesions were cultured on Stonebrinks and Lowenstein-Jensen media at 37°C for eight weeks to detect *M. bovis *[57]. The eight non-infected control animals were 2–3 year old unrelated females selected from a Holstein Friesian herd without a recent history of tuberculosis and were SICTT and IFN-γ test negative. This control animal group also underwent comprehensive testing for the following infections: Brucellosis (*Brucella abortus*), Johne's disease (*Mycobacterium avium *subsp. *paratuberculosis*), infectious bovine rhinotracheitis (bovine herpesvirus-1), salmonella (*Salmonella typhimurium*) and bovine viral diarrhoea (*Pestivirus*).

### Blood sampling and analysis

400 ml of blood was collected from each animal in sterile heparinised bottles. Five ml of blood was used for haematological analysis and leukocyte cell population subsets were compared between infected and control groups as previously described [24].

### PBMC separation, culture, RNA extraction and quality control

PBMC were isolated using the Percoll™ gradient method as previously described [58]. PBMC were seeded at 10^7 ^per flask and cultured in RPMI 1640 culture medium supplemented with 5% FBS, 0.1% mercaptoethanol and 0.1% gentamicin. A total of 48 tissue culture flasks represented 16 individual PBMC samples (BTB-infected [*n *= 8] and healthy control [*n *= 8]) per time point at 0 h (pre-stimulation), 3 h and 12 h post-stimulation with bovine tuberculin. Previous work in our laboratory using PBMC from BTB-infected cattle indicated that these time points would be the most appropriate for further analysis [22]. All PBMC samples were cultured overnight at 37°C in 5% CO_2 _to minimize noise in gene expression measurements potentially introduced by the mechanical disruption of cells associated with PBMC isolation. After cells were harvested for time point 0, remaining flasks were stimulated using 50 μg/ml bovine tuberculin and incubated for 3 h and 12 h at 37°C in 5% CO_2_. Residual cells not seeded for culture in either treatment were immediately dissolved in 3 ml TriReagent^® ^(Molecular Research Centre Inc., Cincinnati, OH) and frozen in 1.5 ml cryotubes at -80°C for use as a common reference RNA (CRR) pool. Total RNA was extracted from PBMC harvested after 3 h and 12 h post-stimulation using a combined TriReagent^®^, DNase treatment and Qiagen RNeasy^® ^method (Qiagen Ltd., Crawley, UK) according to the manufacturers' instructions. The integrity and stability of RNA samples is crucial for gene expression analyses using microarray technology; therefore, RNA yield and quality were assessed using an Agilent 2100 Bioanalyzer (Agilent Technologies Ireland Ltd., Dublin, Ireland). The two-step method for RNA extraction described above was found to produce RNA of high yield and quality (ratios of 18S to 28S ribosomal RNA averaged > 1.6).

### Microarray experimental design

The 3,888 feature BOTL-5 immunogenetic microarray system used has been described previously [59]. The NCBI GEO platform accession for the BOTL-5 microarray is GPL5751. The immunobiology-targeted BOTL-5 array contains 1,391 genes or ESTs spotted in duplicate with multiple additional control features (blank spots, negative spots, housekeeper genes) and is an expanded version of the BOTL-4 array described previously by our group [60,22]. A reference design was used for microarray hybridizations, such that all RNA samples were labelled using Cy3 and co-hybridized with Cy5 labelled common reference RNA (CRR) pool as described previously [24]. Thirty-six arrays were hybridized in total, representing six individual animal PBMC samples from each treatment group pre-stimulation, and at 3 h and 12 h post-stimulation with bovine tuberculin, as shown in Fig. 1. It was hypothesized that the CRR pool would display similar mRNA expression levels and gene coverage as the target samples, therefore allowing flexible, accurate and consistent comparison of gene expression data across a time course without arbitrarily pairing animals from different groups [61]. The CRR pool contained equal amounts of total RNA from the treated and control animal groups.

### cDNA labelling, hybridisation and scanning

cDNA synthesis, Cy3 and Cy5 labelling and microarray hybridizations were performed as previously described [24]. Each labelling reaction contained a total of 8 μg total RNA per sample and 10 μg total RNA from the CRR. Purified labelled samples were combined (either an infected or a control sample combined with a CRR sample) and co-hybridized on the BOTL-5 microarrays using SlideHyb Glass Array Hybridization Buffer #3 (Ambion Ltd.). Microarray hybridizations were performed using a Tecan HS400 hybridisation station (Tecan Ltd.) with the protocol as previously described [24]. Microarrays were scanned immediately using a GenePix 4000B microarray scanner (Molecular Devices Ltd.).

### Data processing, normalization and analysis

The working signal intensities were generated using the mean foreground intensity values minus the median background intensity values as outputted from the GenePix Pro 5.0 results file. Two methods of data pre-processing were used to flag unreliable data as previously described [24].

Median-based normalization, which corrects the data such that all arrays have the same median [62] was used as previously described [24]. Microarray data analysis (including analyses of the microarray-platform specific false discovery rate) was carried out using class comparisons between experimental groups (parametric *t*-tests) as implemented in BRB ArrayTools version 3.0 [63] as previously described [24]. Each gene on the BOTL array is represented by duplicate gene spot features. Analyses of the false discovery rate (FDR) according to methods described previously [24] demonstrated that the differentially expressed gene spot feature lists were robust and reliable. Therefore, to maximise information from these experiments we refer to numbers of differentially expressed gene spot features in the Results section.

### Real time qRT-PCR validation of within group differential gene expression profiles

Replicate spot features on the BOTL array were used as a check for the quality control of gene expression data. Each spot was analyzed individually thereby allowing the individual genes to be flagged if expression results from two or more replicates were statistically different. This enabled the identification of differentially expressed genes that had a low probability of being false positives and expedited the choice of target genes for real time qRT-PCR validation of the microarray results.

The H3 histone family 3A gene (*H3F3A*) was used as a qRT-PCR reference gene for the present study. This gene displayed the least gene expression differences among the 12 control and BTB-infected samples analyzed using the BOTL microarray platform across the time course (data not shown). Gene expression differences detected from total RNA samples from each of the 48 samples (representing eight animals per treatment group) using the BOTL microarray platform were validated using a MX3000P™ fluorescence detection real-time PCR system (Stratagene Europe) as previously described [24]. Gene-specific oligonucleotide primer pairs were designed using Primer Express^® ^version 2.0 software (Applied Biosystems) and synthesized commercially (Invitrogen Ltd.). Experimental details for these primer pairs are shown in Table 4. Further analysis of specific cell regulatory pathways using real-time qRT-PCR concentrated on downstream mediator molecules of TLR signalling to complement those differentially expressed on the microarray. All reactions were performed in duplicate and amplicons for the *H3F3A *reference gene mRNA transcript were used to normalize expression data for the target genes. Real time qRT-PCR data were analysed using the 2^-ΔΔCt ^method [64] within each group, and not between to minimize stochastic error due to cell subpopulation differences between BTB-infected and control animals as previously described [24]. Real time qRT-PCR gene expression log_2 _values from both groups were compared using Student's *t*-test.

**Table 4:** Real time qRT-PCR primer sequences, optimum primer concentrations and amplicon sizes for all validated genes.

**Gene symbol**	**Forward primer (5'-3')**	**Reverse primer (5'-3')**	**Amplicon size (bp)**	**Primer conc. (nM)**
*AKT1*	GGTGATCCTGGTGAAGGAGA	GAGTACTTCAGGGCCGTCAG	159	900
*CHUK*	CCGGAAGCTACTCAACAAACCA	CATGCAAATATCGGATCCCAG	101	900
*H3F3A*	CATGGCTCGTACAAAGCAGA	ACCAGGCCTGTAACGATGAG	136	100
*IFNG*	TGATGGCATGTCAGACAGCA	GGCACAAGTCATATAGCCTGACAC	51	300
*IKBKB*	CCTGAAGATTGCGTGTAGCA	ACTCTGGTCCTGCTCCTTCA	229	300
*IL10*	CTTGTCGGAAATGATCCAGTTTT	TCAGGCCCGTGGTTCTCA	66	300
*IL1A*	GCTATGAGCCACTTCGTGAGGA	TGCCACCATCACCACATTCTC	110	300
*IL1R1*	GAATCCTTTAAACAGAAGAA	TGGATGTATTAGTTGTATGTAT	145	300
*IL1R2*	CCTGTGATCATCTCTTCCCACC	GCAGAGTGGTTGTGTGTATGCC	106	300
*IL1RN*	CCCCACAACCCTTTCATCAA	GGTCAGGAGAAGCCACATTTG	68	300
*IL8*	AGGTGGTGTTTGAAGCCCAT	CACAACCTTCTGCACCCACTT	123	900
*IRAK1*	TCAGCGACTGGACATCCTTCT	GGACGTTGGAACTCTTGACATC	101	300
*LTA*	CCGAGGAGGACTCAGAAACTGA	ACGCCTCTTCTTTCTTCGCCT	111	300
*MAPAPK2*	GGACGTCAAGGAGGAGATGA	CTTCAGAAGCAGAGGGTTGG	106	300
*MYD88*	TGCCTCTGTGTGCCTGTACATC	AGATATGGACCATGGCTGCA	134	300
*NCOR1*	CCTGTGAGAACGAAAACATCAAAC	TTGAGCCTGGTCTCTGATGGT	79	900
*NFKB1*	ATACTGAACAATGCCTTCCGG	CACGTCAATGGCCTCAGTGTAG	135	300
*TGFB1*	TGCTTCAGCTCCACAGAAAAGA	AGGCAGAAATTGGCGTGGT	116	300
*TICAM2*	TGGAGAAGACCCACCTTTGTTT	TAGATCCTCAGCTCTGCTTCGG	163	300
*TLR2*	CCATTGACAAGAAGGCCAT	AACCCTTCCTGCTGAGTCTCAT	106	900
*TLR4*	CGAGAGCACCTATGATGCCTTT	ATGGCCACCCCAGGAATAAA	144	900
*TNF*	TCTACCAGGGAGGAGTCTTCCA	GTCCGGCAGGTTGATCTCA	68	300
*TOLLIP*	AAATGAGAACACAGTGCGCTCT	CATCCCATTAAGCCTACGTGG	186	300

## Authors' contributions

KM was primarily responsible for experimental design, coordination, performance and validation of results. EG and EC provided access to animal samples and valuable expertise in analysis of results. JK and COF provided important comments and discussion as well as manuscript editing. YZ and SP carried out the bioinformatics and microarray data analyses. DM was responsible for experimental design, data analysis, and manuscript preparation and editing. All authors read and approved the final manuscript.

## Supplementary Material

Additional file 1 Table S1, BOTL-5 microarray spot features that showed significant differential expression for the BTB-infected animal group (*n *= 6) between T_3 _and T_0 _(3 h versus 0 h), and between T_12 _and T_3 _(12 h versus 3 h) post-stimulation with PPDb. Green shaded rows detail 250 BOTL-5 microarray spot features that showed significant differential expression for the BTB-infected animal group (*n *= 6) between T_3 _and T_0 _(3 h versus 0 h) post stimulation with bovine tuberculin. Yellow shaded rows detail 80 BOTL-5 microarray spot features that showed significant differential expression for the BTB-infected animal group (*n *= 6) between T_12 _and T_3 _(12 h versus 3 h) post stimulation with bovine tuberculin. [NB. Spot features are ranked by fold-change for each time point comparison]. Click here for file

Additional file 2 Table S2, BOTL-5 microarray spot features that showed significant differential expression for the non-infected control animal group (*n *= 6) between T_3 _and T_0 _(3 h versus 0 h), and between T_12 _and T_3 _(12 h versus 3 h) post-stimulation with PPDb. Green shaded rows detail 88 BOTL-5 microarray spot features that showed significant differential expression for the non-infected control animal group (*n *= 6) between T_3 _and T_0 _(3 h versus 0 h) post stimulation with bovine tuberculin. Yellow shaded rows detail 56 BOTL-5 microarray spot features that showed significant differential expression for the non-infected control animal group (*n *= 6) between T_12 _and T_3 _(12 h versus 3 h) post stimulation with bovine tuberculin. [NB. Spot features are ranked by fold change for each time point comparison]. Click here for file
