# Supplementary material for: Antigen stimulation of peripheral blood mononuclear cells from Mycobacterium bovis infected cattle yields evidence for a novel gene expression program
Source: BMC Genomics. 2008 Sep 29;9:447. doi: 10.1186/1471-2164-9-447 (PMC2569068; doi:10.1186/1471-2164-9-447)
Supplement: Additional file 1 — Table S1, BOTL-5 microarray spot features that showed significant differential expression for the BTB-infected animal group (n = 6) between T3 and T0 (3 h versus 0 h), and between T12 and T3 (12 h versus 3 h) post-stimulation with PPDb. Green shaded rows detail 250 BOTL-5 microarray spot features that showed significant differential expression for the BTB-infected animal group (n = 6) between T3 and T0 (3 h versus 0 h) post stimulation with bovine tuberculin. Yellow shaded rows detail 80 BOTL-5 microarray spot features that showed significant differential expression for the BTB-infected animal group (n = 6) between T12 and T3 (12 h versus 3 h) post stimulation with bovine tuberculin. [NB. Spot features are ranked by fold-change for each time point comparison]. [file 1471-2164-9-447-S1.pdf]

Paper Title: Antigen stimulation of peripheral blood mononuclear cells from *Mycobacterium bovis* infected cattle yields evidence for a novel gene expression program. Meade, K.G. *et al.*

Table S1: Green shaded rows detail 250 BOTL-5 microarray spot features that showed significant differential expression for the BTB-infected animal group (*n* = 6) between T_3 and T_0 (3h versus 0h) post stimulation with bovine tuberculin. Yellow shaded rows detail 80 BOTL-5 microarray spot features that showed significant differential expression for the BTB-infected animal group (*n* = 6) between T_{12} - and T_3 (12h versus 3h) post stimulation with bovine tuberculin. [NB. Spot features are ranked by fold-change for each time point comparison].

Rank	BOTL-5 coordinates	Feature description	Gene symbol	*P*-value	Fold-change	Time point comparison
1	A8.c6	MHC class I heavy chain isoform 1	*BOLA*	0.0004015	5.58	3h vs 0h
2	A8.g9	MHC class I heavy chain isoform 1	*BOLA*	0.0007032	5.28	3h vs 0h
3	C8.g9	similar to chemokine CCL1/I-309 | similar to chemokine CCL1/I-309		0.0008610	5.28	3h vs 0h
4	C8.c6	similar to chemokine CCL1/I-309 | similar to chemokine CCL1/I-309		0.0011065	4.98	3h vs 0h
5	C11.g5	hypothetical protein	*LOC512361*	0.0012870	3.94	3h vs 0h
6	C6.c6	similar to GMCSFR receptor	*LOC511847*	0.0052822	3.86	3h vs 0h
7	D11.i3	Similar to serine/threonine protein kinase	*LOC514210*	0.0010479	3.82	3h vs 0h
8	D11.e1	Similar to serine/threonine protein kinase	*LOC514210*	0.0005247	3.78	3h vs 0h
9	C11.c2	hypothetical protein	*LOC512361*	0.0028230	3.73	3h vs 0h
10	C6.g9	similar to GMCSFR receptor	*LOC511847*	0.0062652	3.65	3h vs 0h
11	B2.b5	translocating chain-associating membrane protein	*TRAM*	0.0029163	3.08	3h vs 0h
12	B2.f8	translocating chain-associating membrane protein	*TRAM*	0.0041705	3.03	3h vs 0h
13	C1.c4	similar to MAP kinase kinase 7 gamma 1	*LOC787278*	0.0023537	3.01	3h vs 0h

Rank	BOTL-5 coordinates	Feature description	Gene symbol	*P*-value	Fold-change	Time point comparison
14	B1.i7	GATA binding protein 4 (GATA4)		0.0073891	2.77	3h vs 0h
15	D11.i4	Transcribed locus, weakly similar to XP_001083311.1 similar to kinase suppressor of ras 2 [Macaca mulatta]		0.0002696	2.75	3h vs 0h
16	A3.c6	CD14 molecule	*CD14*	0.0365308	2.63	3h vs 0h
17	B3.e3	splicing factor, arginine/serine-rich 2	*SFRS2*	0.0011942	2.56	3h vs 0h
18	B3.i5	splicing factor, arginine/serine-rich 2	*SFRS2*	0.0052018	2.47	3h vs 0h
19	B1.e6	GATA binding protein 4 (GATA4)		0.0117014	2.47	3h vs 0h
20	B9.e1	v-akt murine thymoma viral oncogene homolog 1	*AKT1*	0.0065464	2.46	3h vs 0h
21	B3.i8	ephrin-A2	*ENFNA2*	0.0405087	2.46	3h vs 0h
22	D1.e4	GATA-binding protein 4 (GATA4)		0.0046093	2.42	3h vs 0h
23	D9.i4	Sin3A-associated protein, 18kDa	*SAP18*	0.0453981	2.39	3h vs 0h
24	D1.d9	DNA fragmentation factor, 45kDa, alpha polypeptide	*DFFA*	0.0189222	2.37	3h vs 0h
25	B6.e1	eukaryotic translation elongation factor 1 gamma	*EEF1G*	0.0003536	2.36	3h vs 0h
26	D5.e8	nuclear factor of kappa light polypeptide gene enhancer in B-cells 1 (p105)	*NFKB1*	0.0117779	2.34	3h vs 0h
27	A3.g9	CD14 molecule	*CD14*	0.0300464	2.33	3h vs 0h
28	D1.i6	GATA-binding protein 4 (GATA4)		0.0024533	2.30	3h vs 0h
29	B9.i3	v-akt murine thymoma viral oncogene homolog 1	*AKT1*	0.0082823	2.30	3h vs 0h
30	D5.a3	nuclear factor of kappa light polypeptide gene enhancer in B-cells 1 (p105)	*NFKB1*	0.0092788	2.30	3h vs 0h
31	B6.i4	eukaryotic translation elongation factor 2	*EEF2*	0.0000402	2.29	3h vs 0h
32	D2.i2	similar to FGFR1 protein isoform 2	*FGFR1*	0.0150168	2.27	3h vs 0h

Rank	BOTL-5 coordinates	Feature description	Gene symbol	*P*-value	Fold-change	Time point comparison
33	D2.d9	similar to FGFR1 protein isoform 2	*FGFR1*	0.0152872	2.26	3h vs 0h
34	B6.e2	eukaryotic translation elongation factor 2	*EEF2*	0.0000182	2.22	3h vs 0h
35	D3.e6	ephrin-A2 (EFNA2)		0.0077044	2.21	3h vs 0h
36	C5.e8	similar to Tropomyosin 3	*TPM3*	0.0013279	2.16	3h vs 0h
37	D9.i6	v-akt murine thymoma viral oncogene homolog 2	*AKT2*	0.0146746	2.16	3h vs 0h
38	C1.g6	similar to MAP kinase kinase 7 gamma 1	*LOC787278*	0.0101666	2.15	3h vs 0h
39	D11.e2	Transcribed locus, weakly similar to XP_001083311.1 similar to kinase suppressor of ras 2 [Macaca mulatta]		0.0221975	2.14	3h vs 0h
40	B1.h9	Rho guanine nucleotide exchange factor, mRNA (GEF 1)		0.0002962	2.13	3h vs 0h
41	D1.e6	similar to Serum response factor (SRF)	*LOC533039*	0.0008925	2.13	3h vs 0h
42	B6.i3	eukaryotic translation elongation factor 1 gamma	*EEF1G*	0.0010412	2.13	3h vs 0h
43	D9.i8	protein kinase, cAMP-dependent, regulatory, type I, beta	*PRKAR2B*	0.0077058	2.13	3h vs 0h
44	D9.e7	protein kinase, cAMP-dependent, regulatory, type I, beta	*PRKAR2B*	0.0046764	2.12	3h vs 0h
45	A8.f5	similar to Tyrosine-protein kinase Lyn	*LOC534996*	0.0092129	2.12	3h vs 0h
46	C2.g4	TAF6 RNA polymerase II, TATA box binding protein (TBP)-associated factor, 80kDa	*TAF6*	0.0467477	2.12	3h vs 0h
47	D3.i7	ephrin-A2 (EFNA2)		0.0148555	2.11	3h vs 0h
48	C5.a3	similar to Tropomyosin 3	*TPM3*	0.0016879	2.09	3h vs 0h
49	D6.b8	Transcribed locus		0.0043936	2.09	3h vs 0h
50	D6.f1	DAZ associated protein 2	*DAZAP2*	0.0123686	2.09	3h vs 0h
51	D6.g2	Transcribed locus		0.0187316	2.09	3h vs 0h

Rank	BOTL-5 coordinates	Feature description	Gene symbol	*P*-value	Fold-change	Time point comparison
52	B3.i2	mylein basic protein (MBP)		0.0269513	2.09	3h vs 0h
53	A11.b8	similar to death receptor 3	*LOC783432*	0.0153166	2.08	3h vs 0h
54	D12.i8	GAPDH	*GAPDH*	0.0232446	2.07	3h vs 0h
55	B6.d9	actin, beta	*ACTB*	0.0187244	2.06	3h vs 0h
56	D12.h1	GAPDH	*GAPDH*	0.0379525	2.06	3h vs 0h
57	C6.h7	nuclear factor of kappa light polypeptide gene enhancer in B-cells 1 (p105)	*NFKB1*	0.0380419	2.06	3h vs 0h
58	A1.f3	Similar to KIAA0668 protein	*LOC618392*	0.0000731	2.05	3h vs 0h
59	B6.i2	actin, beta	*ACTB*	0.0209279	2.05	3h vs 0h
60	B2.e8	hypothetical protein	*LOC510651*	0.0044301	2.04	3h vs 0h
61	B1.d8	Rho guanine nucleotide exchange factor, mRNA (GEF 1)		0.0016250	2.01	3h vs 0h
62	A11.a3	glioma tumor suppressor candidate region gene 2	*GLTSCR2*	0.0060427	2.01	3h vs 0h
63	D11.i6	insulin receptor substrate 1, mRNA (IRS1)		0.0078117	2.01	3h vs 0h
64	D11.i2	similar to p70 ribosomal S6 kinase beta	*LOC506083*	0.0174021	2.01	3h vs 0h
65	D1.i7	similar to Serum response factor (SRF)	*LOC533039*	0.0016012	2.00	3h vs 0h
66	D6.a5	DAZ associated protein 2	*DAZAP2*	0.0169674	2.00	3h vs 0h
67	B11.d8	SWI/SNF related, matrix associated, actin dependent regulator of chromatin, subfamily a-like 1	*SMARCAL1*	0.0011546	1.98	3h vs 0h
68	B2.d9	fibroblast growth factor receptor 2	*FGFR2*	0.0159178	1.98	3h vs 0h
69	A11.g2	similar to death receptor 3	*LOC783432*	0.0162147	1.98	3h vs 0h
70	D6.i2	similar to neuropilin-1	*LOC539369*	0.0349684	1.98	3h vs 0h

Rank	BOTL-5 coordinates	Feature description	Gene symbol	*P*-value	Fold-change	Time point comparison
71	B3.d9	mylein basic protein (MBP)		0.0434810	1.98	3h vs 0h
72	C4.d9	inhibitor of kappa light polypeptide gene enhancer in B-cells, kinase beta	*IKBKB*	0.0437243	1.98	3h vs 0h
73	A11.e8	glioma tumor suppressor candidate region gene 2	*GLTSCR2*	0.0136695	1.95	3h vs 0h
74	C11.b3	BOTL0100003XC06R	*AY957499*	0.0091897	1.92	3h vs 0h
75	B9.d8	similar to nuclear receptor co-repressor 1 isoform 3	*NCOR1*	0.0113368	1.90	3h vs 0h
76	B9.h9	similar to nuclear receptor co-repressor 1 isoform 3	*NCOR1*	0.0275800	1.90	3h vs 0h
77	A7.f1	uncoupling protein 2 (mitochondrial, proton carrier)	*UCP2*	0.0080319	1.89	3h vs 0h
78	A1.a7	Similar to KIAA0668 protein	*LOC618392*	0.0031005	1.88	3h vs 0h
79	D11.e3	lymphotoxin alpha	*LTA*	0.0226303	1.88	3h vs 0h
80	B6.f2	similar to Rho-related GTP-binding protein Rho6 (Rho family GTPase 1) (Rnd1)		0.0225457	1.87	3h vs 0h
81	A7.g1	leukocyte receptor cluster (LRC) member 1	*LENG1*	0.0068147	1.86	3h vs 0h
82	A8.a8	similar to Tyrosine-protein kinase Lyn	*LOC534996*	0.0023238	1.85	3h vs 0h
83	B2.i2	fibroblast growth factor receptor 2	*FGFR2*	0.0023800	1.84	3h vs 0h
84	B9.e2	similar to C-terminal binding protein 1	*LOC513131*	0.0175062	1.83	3h vs 0h
85	D8.b7	similar to HCF, C1, VCAF, CFF=VP16 accessory protein host cell factor	*LOC512908*	0.0011176	1.82	3h vs 0h
86	A11.f2	similar to Lymphocyte antigen Ly-6E precursor (Retinoic acid-induced gene E protein) (RIG-E) (Thymic shared antigen 1) (TSA-1) (Stem cell antigen 2) (SCA-2)	*SCA-2*	0.0046485	1.77	3h vs 0h
87	A6.b8	similar to SET binding factor 1	*LOC510349*	0.0054413	1.77	3h vs 0h
88	C6.d7	nuclear factor of kappa light polypeptide gene enhancer in B-cells 1 (p105)	*NFKB1*	0.0334521	1.77	3h vs 0h

Rank	BOTL-5 coordinates	Feature description	Gene symbol	*P*-value	Fold-change	Time point comparison
89	A8.i3	similar to MAP kinase-activated protein kinase 2 (MAPK-activated protein kinase 2) (MAPKAP kinase 2) (MAPKAPK-2) (MK2)	*LOC788091*	0.0426963	1.77	3h vs 0h
90	B8.e1	RAS p21 protein activator (GTPase activating protein) 3 (Ins(1,3,4,5)P4-binding protein)	*RASA3*	0.0069693	1.76	3h vs 0h
91	A4.i8	hypothetical protein	*LOC513497*	0.0146517	1.76	3h vs 0h
92	D8.g1	similar to HCF, C1, VCAF, CFF=VP16 accessory protein host cell factor	*LOC512908*	0.0042541	1.75	3h vs 0h
93	D11.d9	similar to p70 ribosomal S6 kinase beta	*LOC506083*	0.0053191	1.75	3h vs 0h
94	A9.e9	hypothetical protein	*LOC514346*	0.0099714	1.75	3h vs 0h
95	C11.f7	BOTL0100003XC06R		0.0123586	1.75	3h vs 0h
96	B6.b7	similar to Syntaxin-5		0.0346125	1.74	3h vs 0h
97	B8.i3	RAS p21 protein activator (GTPase activating protein) 3 (Ins(1,3,4,5)P4-binding protein)	*RASA3*	0.0148152	1.72	3h vs 0h
98	A11.a6	similar to Lymphocyte antigen Ly-6E precursor (Retinoic acid-induced gene E protein) (RIG-E) (Thymic shared antigen 1) (TSA-1) (Stem cell antigen 2) (SCA-2)	*SCA-2*	0.0013375	1.71	3h vs 0h
99	B5.i2	NBFGC_AW659262 BRCA1associated protein-1 (ubiqutin carboxy-		0.0068029	1.71	3h vs 0h
100	A4.e9	similar to SFPQ protein	*LOC618226*	0.0050394	1.70	3h vs 0h
101	A6.a5	similar to actin-binding LIM protein 1 isoform 3	*LOC504968*	0.0169954	1.70	3h vs 0h
102	A3.i6	monocyte-chemoattractant protein-1 precursor	*CCL2*	0.0280098	1.70	3h vs 0h
103	B9.a3	similar to TLE3 protein	*LOC514326*	0.0018475	1.69	3h vs 0h
104	A9.a4	hypothetical protein	*LOC514346*	0.0119533	1.69	3h vs 0h
105	A10.a3	similar to G protein-coupled receptor TGR7	*LOC507247*	0.0006034	1.68	3h vs 0h
106	B9.e8	similar to TLE3 protein	*LOC514326*	0.0032038	1.68	3h vs 0h

Rank	BOTL-5 coordinates	Feature description	Gene symbol	*P*-value	Fold-change	Time point comparison
107	B1.f8	similar to Cathepsin W isoform 2	*LOC510967*	0.0208599	1.68	3h vs 0h
108	D5.e1	prohibitin 2	*PHB2*	0.0339694	1.68	3h vs 0h
109	C5.g5	pyruvate kinase 3	*PKM3*	0.0083753	1.67	3h vs 0h
110	D11.i5	lymphotoxin alpha	*LTA*	0.0037350	1.65	3h vs 0h
111	A8.e1	similar to MAP kinase-activated protein kinase 2 (MAPK-activated protein kinase 2) (MAPKAP kinase 2) (MAPKAPK-2) (MK2)	*LOC788091*	0.0042469	1.65	3h vs 0h
112	A1.g8	endonuclease domain containing 1	*ENDOD1*	0.0296320	1.65	3h vs 0h
113	A2.b5	similar to mitogen-activated protein kinase kinase kinase kinase 2	*LOC520058*	0.0055596	1.64	3h vs 0h
114	A4.a4	similar to SFPQ protein	*LOC618226*	0.0202767	1.63	3h vs 0h
115	D4.g1	hypothetical protein	*LOC786280*	0.0077852	1.62	3h vs 0h
116	C2.c1	TAF6 RNA polymerase II, TATA box binding protein (TBP)-associated factor, 80kDa	*TAF6*	0.0226052	1.62	3h vs 0h
117	A2.f8	similar to mitogen-activated protein kinase kinase kinase kinase 2	*LOC520058*	0.0004584	1.61	3h vs 0h
118	D8.e2	similar to GCN5 general control of amino-acid synthesis 5-like 2 (yeast)	*LOC514420*	0.0010109	1.61	3h vs 0h
119	A9.c4	hypothetical protein	*LOC615329*	0.0137242	1.61	3h vs 0h
120	D6.e1	Unknown	-	0.0150092	1.61	3h vs 0h
121	D8.d3	ADP-ribosyltransferase (NAD+) poly (ADP-ribose) polymerase)	*PARP1*	0.0334735	1.61	3h vs 0h
122	B7.e7	mitogen-activated protein kinase kinase 1, RNA (MAP2K1)		0.0036071	1.59	3h vs 0h
123	C5.c2	pyruvate kinase 3	*PKM3*	0.0353435	1.59	3h vs 0h
124	A6.g2	similar to SET binding factor 1	*LOC510349*	0.0315143	1.58	3h vs 0h
125	B2.a3	hypothetical protein	*LOC510651*	0.0279914	1.57	3h vs 0h

Rank	BOTL-5 coordinates	Feature description	Gene symbol	*P*-value	Fold-change	Time point comparison
126	A9.g6	hypothetical protein	*LOC615329*	0.0158345	1.56	3h vs 0h
127	C2.e2	retinoid X receptor, beta	*RXRB*	0.0003208	1.55	3h vs 0h
128	B3.i6	similar to p101 protein	*LOC614838*	0.0024366	1.53	3h vs 0h
129	A1.c1	similar to AF-4	*LOC781149*	0.0024641	1.53	3h vs 0h
130	C9.b7	similar to Eif5 protein isoform 3	*LOC504752*	0.0486630	1.53	3h vs 0h
131	B3.e4	similar to p101 protein	*LOC614838*	0.0094611	1.52	3h vs 0h
132	B1.b5	similar to Cathepsin W isoform 2	*LOC510967*	0.0097576	1.50	3h vs 0h
133	D9.g1	seryl-tRNA synthetase 2	*SARS2*	0.0429487	1.50	3h vs 0h
134	A1.c5	endonuclease domain containing 1	*ENDOD1*	0.0313649	1.49	3h vs 0h
135	D6.f8	BOTL0100009_A01		0.0135001	1.48	3h vs 0h
136	A8.g2	BOTL0100012_E05		0.0428093	1.48	3h vs 0h
137	A6.f5	similar to neurogranin		0.0409134	1.47	3h vs 0h
138	A9.f3	hypothetical protein	*LOC509987*	0.0232827	1.46	3h vs 0h
139	A6.a8	similar to neurogranin		0.0277665	1.46	3h vs 0h
140	A6.g3	hypothetical protein	*LOC506974*	0.0107055	1.44	3h vs 0h
141	D6.b5	BOTL0100009_A01		0.0117410	1.44	3h vs 0h
142	A11.g5	adenylate cyclase 7	*ADCY7*	0.0212583	1.44	3h vs 0h
143	C4.a4	similar to beta-1,2-N-acetylglucosaminyltransferase II	*LOC790576*	0.0337149	1.44	3h vs 0h
144	A3.f2	Similar to Hexokinase 3 (white cell)	*HK3*	0.0239656	1.43	3h vs 0h

Rank	BOTL-5 coordinates	Feature description	Gene symbol	*P*-value	Fold-change	Time point comparison
145	A9.a7	hypothetical protein	*LOC509987*	0.0189125	1.42	3h vs 0h
146	D4.b7	hypothetical protein	*LOC786280*	0.0045725	1.41	3h vs 0h
147	B9.b6	similar to human immunity associated protein 1	*LOC615733*	0.0481828	1.40	3h vs 0h
148	B4.d3	chemokine (C-C motif) ligand 3-like 1	*CCL3L1*	0.0033673	1.39	3h vs 0h
149	D8.g3	hypothetical protein	*LOC616055*	0.0129208	1.38	3h vs 0h
150	C4.e9	similar to beta-1,2-N-acetylglucosaminyltransferase II	*LOC790576*	0.0131187	1.37	3h vs 0h
151	A6.b9	hypothetical protein		0.0163643	1.37	3h vs 0h
152	C2.f1	Unknown		0.0229631	1.37	3h vs 0h
153	A6.f7	similar to Poly [ADP-ribose] polymerase 10 (PARP-10)	*LOC510991*	0.0035098	1.35	3h vs 0h
154	D6.g1	BOTL0100013_F10	*LOC516783*	0.0354595	1.35	3h vs 0h
155	D8.i4	similar to GCN5 general control of amino-acid synthesis 5-like 2 (yeast)	*LOC514420*	0.0318256	1.34	3h vs 0h
156	D5.a7	strongly similar to XP_001101736.1 similar to homolog of yeast mRNA transport regulator 3 [Macaca mulatta]		0.0275627	1.32	3h vs 0h
157	A4.b9	hypothetical protein	*LOC507577*	0.0339311	1.30	3h vs 0h
158	D3.i8	similar to caspase-2	*CASP2*	0.0327964	1.28	3h vs 0h
159	D8.b9	hypothetical protein	*LOC616055*	0.0075793	1.27	3h vs 0h
160	D8.g2	BOTL0100013_G08		0.0385722	1.26	3h vs 0h
161	D11.c1	hypothetical protein	*LOC532712*	0.0412314	1.25	3h vs 0h
162	B5.a7	similar to gigaxonin	*GAN*	0.0320668	1.24	3h vs 0h
163	D10.b5	BOTL0100009_B10		0.0342398	1.19	3h vs 0h

Rank	BOTL-5 coordinates	Feature description	Gene symbol	*P*-value	Fold-change	Time point comparison
164	D6.i4	similar to calcium/calmodulin-dependent protein kinase I		0.0375180	1.18	3h vs 0h
165	C9.a3	similar to Dynein intermediate chain 2, cytosolic (DH IC-2) (Cytoplasmic dynein intermediate chain 2)	*DYNC1I2*	0.0461438	-1.17	3h vs 0h
166	B8.e9	similar to Kremen2 protein	*LOC618527*	0.0034077	-1.20	3h vs 0h
167	A8.e9	hypothetical protein	*LOC768044*	0.0101607	-1.20	3h vs 0h
168	C5.g2	Transcribed locus		0.0173350	-1.22	3h vs 0h
169	A10.g2	similar to tangerin C	*LOC788760*	0.0268463	-1.23	3h vs 0h
170	C8.g5	similar to Interleukin-1 receptor-associated kinase 4 (IRAK-4) (NY-REN-64 antigen)		0.0441493	-1.24	3h vs 0h
171	D11.g6	BOTL0100012_A06		0.0327683	-1.25	3h vs 0h
172	B5.a6	similar to protein tyrosine phosphatase, receptor type, F	*LOC790379*	0.0392893	-1.26	3h vs 0h
173	C11.a3	similar to ZNF786 protein	*LOC506408*	0.0098941	-1.27	3h vs 0h
174	D8.f3	hypothetical protein	*LOC617688*	0.0278973	-1.28	3h vs 0h
175	C3.f3	ribosomal protein, large, P1	*RPLP1*	0.0248478	-1.29	3h vs 0h
176	B7.b3	BOTL0100004XF08R		0.0325903	-1.29	3h vs 0h
177	A1.e9	hypothetical protein	*LOC537453*	0.0487361	-1.29	3h vs 0h
178	D11.f3	ret finger protein 2	*TRIM13*	0.0210337	-1.31	3h vs 0h
179	C3.a7	ribosomal protein, large, P1	*RPLP1*	0.0293211	-1.31	3h vs 0h
180	A10.b8	similar to tangerin C	*LOC788760*	0.0297898	-1.31	3h vs 0h
181	B10.b7	splicing factor, arginine/serine-rich 7, 35kDa	*SFRS7*	0.0462638	-1.32	3h vs 0h
182	C1.a3	protein phosphatase 2 (formerly 2A), catalytic subunit, alpha isoform	*PPP2R2A*	0.0154861	-1.33	3h vs 0h

Rank	BOTL-5 coordinates	Feature description	Gene symbol	*P*-value	Fold-change	Time point comparison
183	C4.a7	similar to Pre-mRNA cleavage complex 2 protein Pcf11 (Pre-mRNA cleavage complex II protein Pcf11)	*LOC510604*	0.0253967	-1.33	3h vs 0h
184	C4.a8	guanine nucleotide binding protein (G protein), alpha inhibiting activity polypeptide 3	*GNAI3*	0.0332849	-1.33	3h vs 0h
185	C5.e9	similar to Serine/threonine protein phosphatase 6 (PP6)	*LOC608922*	0.0436579	-1.33	3h vs 0h
186	B9.f7	BOTL0100004XG08R		0.0031473	-1.34	3h vs 0h
187	D10.a3	Transcribed locus		0.0135365	-1.34	3h vs 0h
188	A10.f7	similar to insulin induced gene 1		0.0476418	-1.34	3h vs 0h
189	C3.g3	BOTL0100009_E08		0.0036865	-1.36	3h vs 0h
190	B10.a4	hypothetical protein	*LOC515972*	0.0045413	-1.36	3h vs 0h
191	A6.i5	similar to AXL receptor tyrosine kinase	*LOC516598*	0.0115676	-1.36	3h vs 0h
192	D11.a7	ret finger protein 2	*TRIM13*	0.0151925	-1.36	3h vs 0h
193	C8.g3	BOTL0100012_E09		0.0482115	-1.36	3h vs 0h
194	B10.g1	splicing factor, arginine/serine-rich 7, 35kDa	*SFRS7*	0.0035702	-1.38	3h vs 0h
195	B10.e9	hypothetical protein	*LOC515972*	0.0312184	-1.38	3h vs 0h
196	D5.e9	BOTL0100003XF04R	-	0.0469598	-1.38	3h vs 0h
197	D4.e9	nuclear factor (erythroid-derived 2)-like 2	*NFE2L2*	0.0349485	-1.39	3h vs 0h
198	C9.e3	chemokine (C-X-C motif) ligand 2	*CXCL2*	0.0418240	-1.39	3h vs 0h
199	C10.g1	similar to ryanodine receptor 3 | similar to cell death regulator aven	*LOC539899 | LOC512666*	0.0367274	-1.40	3h vs 0h
200	B9.b3	BOTL0100004XG08R		0.0012430	-1.41	3h vs 0h
201	C9.a6	similar to Polyadenylate-binding protein-interacting protein 2 (Poly(A) binding protein-interacting protein 2) (PABP-interacting protein 2)	*PAIP-2*	0.0350569	-1.41	3h vs 0h

Rank	BOTL-5 coordinates	Feature description	Gene symbol	*P*-value	Fold-change	Time point comparison
		(PAIP-2)				
202	A6.c5	adaptor-related protein complex 1, sigma 1 subunit	*AP1S1*	0.0129807	-1.42	3h vs 0h
203	C10.c8	similar to subtilisin-like proprotein convertase (EC 3.4.21.-) PACE4 precursor, splice form A - human	*LOC524684*	0.0160122	-1.42	3h vs 0h
204	A12.g6	hypothetical protein	*LOC509282*	0.0089175	-1.43	3h vs 0h
205	C9.f2	similar to Polyadenylate-binding protein-interacting protein 2 (Poly(A) binding protein-interacting protein 2) (PABP-interacting protein 2) (PAIP-2)	*PAIP-2*	0.0254984	-1.43	3h vs 0h
206	B11.e8	hydroxysteroid (17-beta) dehydrogenase 4	*HSD17B4*	0.0358533	-1.43	3h vs 0h
207	D10.e8	Transcribed locus		0.0025625	-1.44	3h vs 0h
208	D11.e6	Transcribed locus, strongly similar to NP_005345.3 D proto-oncogene		0.0161487	-1.44	3h vs 0h
209	B9.f5	BOTL0100004XG06R		0.0309670	-1.44	3h vs 0h
210	A3.e8	H3 histone, family 3A	*H3F3A*	0.0320312	-1.44	3h vs 0h
211	B10.b6	hypothetical protein	*LOC788514*	0.0151206	-1.45	3h vs 0h
212	C6.e9	BOTL0100006XA10R		0.0403011	-1.45	3h vs 0h
213	C3.f8	Ribosomal protein S3Ae	*RPS3AE*	0.0213061	-1.46	3h vs 0h
214	B10.f1	small inducible cytokine subfamily E, member 1	*SCYE1*	0.0266269	-1.46	3h vs 0h
215	C10.g8	similar to leukocyte differentiation antigen CD84	*LOC510910*	0.0385931	-1.47	3h vs 0h
216	A10.b3	similar to insulin induced gene 1		0.0148179	-1.48	3h vs 0h
217	D5.c2	Transcribed locus		0.0494607	-1.48	3h vs 0h
218	B9.g3	Transcribed locus		0.0044560	-1.49	3h vs 0h
219	C10.h2	similar to subtilisin-like proprotein convertase (EC 3.4.21.-) PACE4	*LOC524684*	0.0151789	-1.49	3h vs 0h

Rank	BOTL-5 coordinates	Feature description	Gene symbol	*P*-value	Fold-change	Time point comparison
		precursor, splice form A - human				
220	A5.f8	hypothetical protein	*LOC617051*	0.0434455	-1.49	3h vs 0h
221	A12.c4	hypothetical protein	*LOC509282*	0.0019617	-1.50	3h vs 0h
222	C5.a4	similar to Serine/threonine protein phosphatase 6 (PP6)	*LOC608922*	0.0263874	-1.50	3h vs 0h
223	B10.f9	hypothetical protein	*LOC788514*	0.0119391	-1.51	3h vs 0h
224	C3.b5	Ribosomal protein S3Ae	*RPS3AE*	0.0132482	-1.51	3h vs 0h
225	B4.i4	hypothetical protein isoform 4	*LOC617051*	0.0393397	-1.51	3h vs 0h
226	B5.g4	similar to antigen identified by monoclonal antibody Ki-67	*LOC513220*	0.0045181	-1.52	3h vs 0h
227	C1.e8	protein phosphatase 2 (formerly 2A), catalytic subunit, alpha isoform	*PPP2R2A*	0.0012290	-1.55	3h vs 0h
228	A10.c1	similar to jumonji domain containing 1C	*LOC781067*	0.0093736	-1.55	3h vs 0h
229	C2.c2	ribosomal protein L6	*RPL6*	0.0260173	-1.55	3h vs 0h
230	B9.b9	Transcribed locus		0.0454385	-1.55	3h vs 0h
231	B11.f2	similar to NIR2	*LOC533859*	0.0377963	-1.56	3h vs 0h
232	D5.g5	Transcribed locus		0.0123478	-1.57	3h vs 0h
233	A4.i5	hypothetical protein	*LOC786378*	0.0160048	-1.58	3h vs 0h
234	B11.a8	Wiskott-Aldrich syndrome protein interacting protein	*WIPF1*	0.0136073	-1.59	3h vs 0h
235	C11.e7	similar to peptidyl-Pro cis trans isomerase isoform 1		0.0497340	-1.59	3h vs 0h
236	B11.e3	Transcribed locus, moderately similar to XP_001056150.1 similar to very large G-protein coupled receptor 1 [Rattus norvegicus]		0.0308682	-1.63	3h vs 0h
237	C7.f3	hypothetical protein	*LOC788861*	0.0387677	-1.63	3h vs 0h

Rank	BOTL-5 coordinates	Feature description	Gene symbol	*P*-value	Fold-change	Time point comparison
238	B11.a6	similar to NIR2	*LOC533859*	0.0263201	-1.64	3h vs 0h
239	A10.g4	similar to jumonji domain containing 1C	*LOC781067*	0.0388252	-1.66	3h vs 0h
240	A9.c2	similar to Conserved oligomeric Golgi complex component 4		0.0071944	-1.67	3h vs 0h
241	C7.a7	hypothetical protein	*LOC788861*	0.0347999	-1.69	3h vs 0h
242	A9.c1	similar to ornithine decarboxylase antizyme 2	*LOC524677*	0.0186440	-1.73	3h vs 0h
243	A5.f2	beta-2-microglobulin	*B2M*	0.0420221	-1.74	3h vs 0h
244	C11.i8	similar to peptidyl-Pro cis trans isomerase isoform 1		0.0414637	-1.76	3h vs 0h
245	A7.g3	syntaxin 7	*STX7*	0.0430864	-1.77	3h vs 0h
246	A9.g5	similar to Conserved oligomeric Golgi complex component 4		0.0249877	-1.78	3h vs 0h
247	A9.b5	similar to carnitine O-palmitoyltransferase	*LOC506812*	0.0044627	-1.79	3h vs 0h
248	B3.a6	similar to methionine-tRNA synthetase	*LOC614580*	0.0061971	-1.81	3h vs 0h
249	D10.f7	hypothetical protein	*LOC515358*	0.0138063	-1.81	3h vs 0h
250	B3.f2	similar to methionine-tRNA synthetase	*LOC614580*	0.0060607	-1.90	3h vs 0h
1	C9.f8	similar to Interferon-induced guanylate-binding protein 1 (GTP-binding protein 1) (Guanine nucleotide-binding protein 1) (HuGBP-1)	*LOC512486*	0.0405088	2.50	12h vs 3h
2	C9.b5	similar to Interferon-induced guanylate-binding protein 1 (GTP-binding protein 1) (Guanine nucleotide-binding protein 1) (HuGBP-1)	*LOC512486*	0.0320301	2.46	12h vs 3h
3	A7.g3	syntaxin 7	*STX7*	0.0296542	1.70	12h vs 3h
4	C5.h4	collagenase	*MMP1*	0.0027474	1.66	12h vs 3h
5	A11.g4	similar to chromosome 17 open reading frame 27	*LOC509283*	0.0334622	1.58	12h vs 3h
6	B9.b9	BOTL0100011_C03		0.0056377	1.52	12h vs 3h

Rank	BOTL-5 coordinates	Feature description	Gene symbol	*P*-value	Fold-change	Time point comparison
7	D12.f1	BOTL0100008_F04		0.0064079	1.41	12h vs 3h
8	C10.h2	similar to subtilisin-like proprotein convertase (EC 3.4.21.-) PACE4 precursor, splice form A - human	*LOC524684*	0.0498153	1.40	12h vs 3h
9	C5.b8	BOTL0100009_F06		0.0447063	1.22	12h vs 3h
10	A10.g2	similar to tangerin C	*LOC788760*	0.0318948	1.10	12h vs 3h
11	C11.g4	BTG family, member 3	*BTG3*	0.0459919	-1.13	12h vs 3h
12	A4.g1	thioredoxin-related transmembrane protein 2	*TMX2*	0.0363280	-1.17	12h vs 3h
13	B5.g3	hypothetical protein	*LOC522360*	0.0450938	-1.20	12h vs 3h
14	C2.f1	Unknown		0.0316986	-1.26	12h vs 3h
15	A2.b6	BOTL0100012_A12		0.0363306	-1.27	12h vs 3h
16	D3.f8	Hypothetical protein	*LOC507199*	0.0062010	-1.28	12h vs 3h
17	B1.f8	similar to Cathepsin W isoform 2	*LOC510967*	0.0351526	-1.29	12h vs 3h
18	C12.a3	abhydrolase domain containing 8	*ABHD8*	0.0052133	-1.31	12h vs 3h
19	C5.c4	similar to notch 2 preproprotein	*LOC513730*	0.0324785	-1.32	12h vs 3h
20	B7.i8	mitogen-activated protein kinase kinase 1, RNA (MAP2K1)		0.0088545	-1.34	12h vs 3h
21	A1.c1	similar to AF-4	*LOC781149*	0.0498954	-1.34	12h vs 3h
22	C5.i3	macrophage migration inhibitory factor (glycosylation-inhibiting factor)	*MIF*	0.0379865	-1.37	12h vs 3h
23	D2.g3	similar to ribosome associated membrane protein 4		0.0341231	-1.38	12h vs 3h
24	D1.i8	similar to SIT protein isoform 1	*LOC512027*	0.0152282	-1.39	12h vs 3h
25	C7.f7	coactosin-like 1 (Dictyostelium)	*COTL1*	0.0336607	-1.39	12h vs 3h

Rank	BOTL-5 coordinates	Feature description	Gene symbol	*P*-value	Fold-change	Time point comparison
26	C2.e2	retinoid X receptor, beta	*RXRB*	0.0050493	-1.40	12h vs 3h
27	B5.i4	Growth hormone	*GH*	0.0368254	-1.46	12h vs 3h
28	B7.e7	mitogen-activated protein kinase kinase 1, RNA (MAP2K1)		0.0025488	-1.47	12h vs 3h
29	D9.b7	seryl-tRNA synthetase 2	*SARS2*	0.0187143	-1.47	12h vs 3h
30	D1.e6	similar to Serum response factor (SRF)	*LOC533039*	0.0482394	-1.48	12h vs 3h
31	D1.i7	similar to Serum response factor (SRF)	*LOC533039*	0.0387187	-1.49	12h vs 3h
32	A3.f2	Similar to Hexokinase 3 (white cell)	*LOC510616*	0.0302245	-1.50	12h vs 3h
33	A1.g8	endonuclease domain containing 1		0.0305252	-1.50	12h vs 3h
34	B1.h9	Rho guanine nucleotide exchange factor, mRNA (GEF 1)		0.0330438	-1.50	12h vs 3h
35	A2.f5	Unknown		0.0452648	-1.50	12h vs 3h
36	C12.a8	Similar to Hexokinase 3 (white cell)	*LOC510616*	0.0057040	-1.51	12h vs 3h
37	A9.a8	CD81 molecule	*CD81*	0.0492306	-1.52	12h vs 3h
38	D4.b7	hypothetical protein	*LOC786280*	0.0439083	-1.55	12h vs 3h
39	D7.e6	nitric oxide synthase 3 (endothelial cell)	*NOS3*	0.0247806	-1.56	12h vs 3h
40	C5.g5	pyruvate kinase 3	*PKM2*	0.0431808	-1.56	12h vs 3h
41	A9.f5	CD81 molecule	*CD81*	0.0309986	-1.57	12h vs 3h
42	A2.f8	similar to mitogen-activated protein kinase kinase kinase kinase 2	*LOC520058*	0.0029827	-1.59	12h vs 3h
43	C2.g4	TAF6 RNA polymerase II, TATA box binding protein (TBP)-associated factor, 80kDa	*TAF6*	0.0306969	-1.60	12h vs 3h
44	A2.b5	similar to mitogen-activated protein kinase kinase kinase kinase 2	*LOC520058*	0.0048977	-1.61	12h vs 3h

Rank	BOTL-5 coordinates	Feature description	Gene symbol	*P*-value	Fold-change	Time point comparison
45	B12.a4	hypothetical protein	*LOC514346*	0.0221045	-1.61	12h vs 3h
46	B12.a7	similar to retinoblastoma-associated factor 600 (RBAF600) isoform 1	*LOC514561*	0.0326070	-1.62	12h vs 3h
47	D4.g1	hypothetical protein	*LOC786280*	0.0123459	-1.64	12h vs 3h
48	B4.b5	similar to cyclic nucleotide phophodiesterase	*LOC533323*	0.0473840	-1.64	12h vs 3h
49	B7.d8	similar to KIAA2014 protein	*LOC540105*	0.0454207	-1.68	12h vs 3h
50	B12.e9	hypothetical protein	*LOC514346*	0.0052202	-1.69	12h vs 3h
51	D1.i6	GATA-binding protein 4 (GATA4)		0.0497362	-1.69	12h vs 3h
52	B11.h9	SWI/SNF related, matrix associated, actin dependent regulator of chromatin, subfamily a-like 1	*SMARCAL1*	0.0110905	-1.71	12h vs 3h
53	B1.e6	GATA binding protein 4 (GATA4)		0.0421441	-1.72	12h vs 3h
54	A8.a8	similar to Tyrosine-protein kinase Lyn	*LOC534996*	0.0485333	-1.75	12h vs 3h
55	B12.f3	similar to retinoblastoma-associated factor 600 (RBAF600) isoform 1	*LOC514561*	0.0068731	-1.78	12h vs 3h
56	B8.i3	RAS p21 protein activator (GTPase activating protein) 3 (Ins(1,3,4,5)P4-binding protein)	*RASA3*	0.0200022	-1.79	12h vs 3h
57	B3.i2	mylein basic protein (MBP)		0.0447012	-1.82	12h vs 3h
58	B1.d8	Rho guanine nucleotide exchange factor, mRNA (GEF 1)		0.0027317	-1.85	12h vs 3h
59	C6.h7	nuclear factor of kappa light polypeptide gene enhancer in B-cells 1 (p105)	*NFKB1*	0.0316886	-1.85	12h vs 3h
60	B6.e1	eukaryotic translation elongation factor 1 gamma	*EEF1G*	0.0256508	-1.87	12h vs 3h
61	D9.i8	protein kinase, cAMP-dependent, regulatory, type I, beta	*PRKAR1B*	0.0454800	-1.87	12h vs 3h
62	B8.e1	RAS p21 protein activator (GTPase activating protein) 3 (Ins(1,3,4,5)P4-binding protein)	*RASA3*	0.0291039	-1.90	12h vs 3h
63	A11.e8	glioma tumor suppressor candidate region gene 2	*GLTSCR2*	0.0314618	-1.99	12h vs 3h

Rank	BOTL-5 coordinates	Feature description	Gene symbol	*P*-value	Fold-change	Time point comparison
64	A11.a3	glioma tumor suppressor candidate region gene 2	*GLTSCR2*	0.0367875	-1.99	12h vs 3h
65	D11.e1	Similar to serine/threonine protein kinase	*LOC514210*	0.0423519	-1.99	12h vs 3h
66	B9.e1	v-akt murine thymoma viral oncogene homolog 1	*AKT1*	0.0377055	-2.01	12h vs 3h
67	A1.a7	Similar to KIAA0668 protein	*LOC618392*	0.0125049	-2.02	12h vs 3h
68	A1.f3	Similar to KIAA0668 protein	*LOC618392*	0.0084437	-2.05	12h vs 3h
69	D11.i3	Similar to serine/threonine protein kinase	*LOC514210*	0.0405599	-2.08	12h vs 3h
70	A2.g6	hypothetical protein	*LOC614093*	0.0007606	-2.16	12h vs 3h
71	A3.g9	CD14 molecule	*CD14*	0.0380259	-2.24	12h vs 3h
72	B3.i5	splicing factor, arginine/serine-rich 2	*SFRS2*	0.0107057	-2.29	12h vs 3h
73	B3.e3	splicing factor, arginine/serine-rich 2	*SFRS2*	0.0113258	-2.39	12h vs 3h
74	C1.c4	similar to MAP kinase kinase 7 gamma 1	*LOC787278*	0.0259424	-2.60	12h vs 3h
75	C8.c6	similar to chemokine CCL1/I-309 | similar to chemokine CCL1/I-309	*LOC786156* | *LOC786204*	0.0234985	-2.65	12h vs 3h
76	C8.g9	similar to chemokine CCL1/I-309 | similar to chemokine CCL1/I-309	*LOC786156* | *LOC786204*	0.0142840	-2.75	12h vs 3h
77	C11.c2	hypothetical protein	*LOC512361*	0.0182371	-2.78	12h vs 3h
78	C11.g5	hypothetical protein	*LOC512361*	0.0213715	-2.82	12h vs 3h
79	A8.g9	MHC class I heavy chain isoform 1	*BOLA*	0.0161226	-2.93	12h vs 3h
80	A8.c6	MHC class I heavy chain isoform 1	*BOLA*	0.0138905	-3.06	12h vs 3h
