# Supplementary material for: Antigen stimulation of peripheral blood mononuclear cells from Mycobacterium bovis infected cattle yields evidence for a novel gene expression program
Source: BMC Genomics. 2008 Sep 29;9:447. doi: 10.1186/1471-2164-9-447 (PMC2569068; doi:10.1186/1471-2164-9-447)
Supplement: Additional file 2 — Table S2, BOTL-5 microarray spot features that showed significant differential expression for the non-infected control animal group (n = 6) between T3 and T0 (3 h versus 0 h), and between T12 and T3 (12 h versus 3 h) post-stimulation with PPDb. Green shaded rows detail 88 BOTL-5 microarray spot features that showed significant differential expression for the non-infected control animal group (n = 6) between T3 and T0 (3 h versus 0 h) post stimulation with bovine tuberculin. Yellow shaded rows detail 56 BOTL-5 microarray spot features that showed significant differential expression for the non-infected control animal group (n = 6) between T12 and T3 (12 h versus 3 h) post stimulation with bovine tuberculin. [NB. Spot features are ranked by fold change for each time point comparison]. [file 1471-2164-9-447-S2.pdf]

Paper Title: Antigen stimulation of peripheral blood mononuclear cells from *Mycobacterium bovis* infected cattle yields evidence for a novel gene expression program. Meade, K.G. *et al.*

Table S2: Green shaded rows detail 88 BOTL-5 microarray spot features that showed significant differential expression for the non-infected control animal group (*n* = 6) between T_3 and T_0 (3h versus 0h) post stimulation with bovine tuberculin. Yellow shaded rows detail 56 BOTL-5 microarray spot features that showed significant differential expression for the non-infected control animal group (*n* = 6) between T_{12} and T_3 (12h versus 3h) post stimulation with bovine tuberculin. [NB. Spot features are ranked by fold change for each time point comparison].

Rank	BOTL-5 coordinates	Feature description	Gene symbol	*P*-value	Fold-change	Time point comparison
1	C1.h4	interleukin 8	*IL8*	0.0339158	2.22	3h vs 0h
2	A6.e3	similar to AXL receptor tyrosine kinase	*LOC516598*	0.0477895	1.93	3h vs 0h
3	D7.i8	mitogen-activated protein kinase 13	*MAPK13*	0.0143389	1.90	3h vs 0h
4	C10.e1	nuclear factor of activated T-cells, cytoplasmic, calcineurin-dependent 4	*NFATC4*	0.0040454	1.83	3h vs 0h
5	D5.i6	similar to casein kinase I-beta	*LOC785423*	0.0366969	1.80	3h vs 0h
6	D7.i5	cyclin B2	*CCNB2*	0.0132994	1.79	3h vs 0h
7	D7.e7	mitogen-activated protein kinase 13	*MAPK13*	0.0321791	1.79	3h vs 0h
8	D9.e3	BF074360 P120 Inhibin receptor, immunoglobulin superfamily-		0.0412172	1.77	3h vs 0h
9	C1.c2	Xylosyltransferase II	*XYLT2*	0.0145205	1.70	3h vs 0h
10	C4.i8	similar to AXL receptor tyrosine kinase	*LOC516598*	0.0484454	1.69	3h vs 0h
11	C3.g1	similar to dynamin 2	*LOC511691*	0.0060188	1.68	3h vs 0h
12	C1.b8	hypothetical protein	*LOC539923*	0.0049735	1.67	3h vs 0h
13	B6.a8	similar to Brain protein I3 (pRGR2)	*LOC615390*	0.0252943	1.66	3h vs 0h

Rank	BOTL-5 coordinates	Feature description	Gene symbol	*P*-value	Fold-change	Time point comparison
14	C2.e4	growth arrest and DNA-damage-inducible, alpha	*GADD45A*	0.0024995	1.64	3h vs 0h
15	A3.b9	Parkinson disease (autosomal recessive, early onset) 7	*PARK7*	0.0042519	1.62	3h vs 0h
16	A1.f2	eukaryotic translation initiation factor 4 gamma, 2	*EIF4G2*	0.0278052	1.61	3h vs 0h
17	D1.f7	N-myc downstream regulated gene 1	*NDRG1*	0.0060494	1.59	3h vs 0h
18	C3.g3	BOTL0100009_E08		0.0369298	1.58	3h vs 0h
19	B9.f5	BOTL0100004XG06R		0.0466285	1.57	3h vs 0h
20	A3.b8	similar to Cell division cycle 34 homolog (S. cerevisiae)	*LOC616156*	0.0162431	1.53	3h vs 0h
21	C2.b5	similar to SCY1-like 3		0.0252009	1.50	3h vs 0h
22	C1.f3	BOTL0100002XD06R		0.0041564	1.48	3h vs 0h
23	D2.b6	hypothetical protein	*LOC537028*	0.0375238	1.48	3h vs 0h
24	C3.f1	similar to protein phosphatase 1, regulatory subunit 9B	*LOC787141*	0.0105992	1.46	3h vs 0h
25	A1.f8	BOTL0100002XE07R		0.0372668	1.46	3h vs 0h
26	C10.a4	similar to Poly [ADP-ribose] polymerase 10 (PARP-10)	*LOC510991*	0.0317206	1.44	3h vs 0h
27	B5.i8	hypothetical protein	*LOC515777*	0.0402354	1.44	3h vs 0h
28	B1.a8	similar to Ecotropic viral integration site 2B		0.0481491	1.44	3h vs 0h
29	C3.a5	similar to protein phosphatase 1, regulatory subunit 9B	*LOC787141*	0.0076385	1.43	3h vs 0h
30	D3.i4	max-associated protein (MAD4)		0.0111948	1.43	3h vs 0h
31	D3.f3	similar to human immunity associated protein 1	*LOC615733*	0.0147195	1.40	3h vs 0h
32	C1.g2	hypothetical protein	*LOC539923*	0.0129359	1.39	3h vs 0h

Rank	BOTL-5 coordinates	Feature description	Gene symbol	*P*-value	Fold-change	Time point comparison
33	A3.g5	O-sialoglycoprotein endopeptidase	*OSGEP*	0.0241653	1.39	3h vs 0h
34	C10.b3	BOTL0100007_D05		0.0243122	1.38	3h vs 0h
35	B5.e8	protein tyrosine phosphatase, receptor type, C-associated protein	*PTPRCAP*	0.0293541	1.37	3h vs 0h
36	A9.a3	tyrosine 3-monooxygenase/tryptophan 5-monooxygenase activation protein, eta polypeptide	*YWHAH*	0.0375958	1.37	3h vs 0h
37	D1.i8	similar to SIT protein isoform 1	*LOC512027*	0.0396894	1.37	3h vs 0h
38	B1.a7	Transcribed locus, strongly similar to XP_001107198.1 cyclin-dependent kinase inhibitor 2B (p15, inhibits CDK4) isoform 1 [Macaca mulatta]		0.0299652	1.35	3h vs 0h
39	B2.i4	similar to Signal transducer and activator of transcription 5B	*LOC789476*	0.0318601	1.34	3h vs 0h
40	D4.b5	Transcribed locus, weakly similar to XP_001368969.1 similar to Kruppel-like zinc finger transcription factor [Monodelphis domestica]		0.0158395	1.24	3h vs 0h
41	B1.f5	similar to Ecotropic viral integration site 2B		0.0096597	1.20	3h vs 0h
42	B8.a3	similar to zinc finger protein 258	*LOC618234*	0.0231149	-1.19	3h vs 0h
43	A6.g4	BOTL0100013_A04		0.0079003	-1.22	3h vs 0h
44	A4.f5	BOTL0100006XF07R		0.0249586	-1.27	3h vs 0h
45	C5.e1	macrophage migration inhibitory factor (glycosylation-inhibiting factor)	*MIF*	0.0312857	-1.27	3h vs 0h
46	A2.h9	(NSD) insulin-like 3, mRNA (INSL3)		0.0344749	-1.27	3h vs 0h
47	B12.a5	hypothetical protein	*LOC523618*	0.0481437	-1.36	3h vs 0h
48	C7.g5	similar to human immunity associated protein 1	*LOC615733*	0.0285923	-1.37	3h vs 0h
49	D4.b7	hypothetical protein	*LOC786280*	0.0021148	-1.38	3h vs 0h
50	B2.a3	hypothetical protein	*LOC510651*	0.0245340	-1.40	3h vs 0h

Rank	BOTL-5 coordinates	Feature description	Gene symbol	*P*-value	Fold-change	Time point comparison
51	C2.c1	TAF6 RNA polymerase II, TATA box binding protein (TBP)-associated factor, 80kDa	*TAF6*	0.0070652	-1.42	3h vs 0h
52	C9.c5	Prolyl endopeptidase	*PREP*	0.0204150	-1.43	3h vs 0h
53	B1.i5	heat shock 27kDa protein 1	*HSPB1*	0.0357928	-1.43	3h vs 0h
54	C8.i4	(NSD) NBFGC_BF773656 SMAD 7 MAD-related gene		0.0336135	-1.46	3h vs 0h
55	B6.e1	eukaryotic translation elongation factor 1 gamma	*EEF1G*	0.0468064	-1.46	3h vs 0h
56	C5.c2	pyruvate kinase 3	*PKM2*	0.0383230	-1.47	3h vs 0h
57	B9.b6	similar to human immunity associated protein 1	*LOC615733*	0.0084612	-1.48	3h vs 0h
58	B9.f9	similar to human immunity associated protein 1	*LOC615733*	0.0055554	-1.51	3h vs 0h
59	D1.e4	GATA-binding protein 4 (GATA4)		0.0145124	-1.51	3h vs 0h
60	C12.g2	hypothetical gene supported by BC069011		0.0163030	-1.51	3h vs 0h
61	B6.b8	similar to echinoderm microtubule-associated protein-like EMAP2	*LOC512380*	0.0425366	-1.51	3h vs 0h
62	C5.i3	macrophage migration inhibitory factor (glycosylation-inhibiting factor)	*MIF*	0.0350640	-1.53	3h vs 0h
63	C6.g2	similar to nucleoporin 214kDa	*LOC784219*	0.0002050	-1.54	3h vs 0h
64	D8.b7	similar to HCF, C1, VCAF, CFF=VP16 accessory protein host cell factor	*LOC512908*	0.0163401	-1.54	3h vs 0h
65	B4.f7	glutathione synthetase	*GSS*	0.0461202	-1.59	3h vs 0h
66	C5.c4	similar to notch 2 preproprotein	*LOC513730*	0.0436370	-1.60	3h vs 0h
67	B1.i7	GATA binding protein 4 (GATA4)		0.0220182	-1.61	3h vs 0h
68	B3.i5	splicing factor, arginine/serine-rich 2	*SFRS2*	0.0465447	-1.61	3h vs 0h
69	C7.a3	hypothetical protein	*LOC529141*	0.0335372	-1.62	3h vs 0h

Rank	BOTL-5 coordinates	Feature description	Gene symbol	*P*-value	Fold-change	Time point comparison
70	B3.e3	splicing factor, arginine/serine-rich 2	*SFRS2*	0.0245861	-1.66	3h vs 0h
71	A2.c7	similar to Neutral sphingomyelinase (N-SMase) activation associated factor	*LOC509626*	0.0369785	-1.66	3h vs 0h
72	C7.b3	coactosin-like 1 (Dictyostelium)	*COTL1*	0.0096828	-1.67	3h vs 0h
73	A8.f5	similar to Tyrosine-protein kinase Lyn	*LOC534996*	0.0260586	-1.70	3h vs 0h
74	A10.f5	nurim (nuclear envelope membrane protein)	*NRM*	0.0179106	-1.75	3h vs 0h
75	A9.e9	hypothetical protein	*LOC514346*	0.0182601	-1.75	3h vs 0h
76	C6.b8	similar to nucleoporin 214kDa	*LOC784219*	0.0056924	-1.76	3h vs 0h
77	B6.g2	similar to echinoderm microtubule-associated protein-like EMAP2	*LOC512380*	0.0101119	-1.79	3h vs 0h
78	B9.a3	similar to TLE3 protein	*LOC514326*	0.0364183	-1.86	3h vs 0h
79	A11.e8	glioma tumor suppressor candidate region gene 2	*GLTSCR2*	0.0256987	-1.92	3h vs 0h
80	A9.a4	hypothetical protein	*LOC514346*	0.0212442	-1.95	3h vs 0h
81	D8.g1	similar to HCF, C1, VCAF, CFF=VP16 accessory protein host cell factor	*LOC512908*	0.0463108	-1.97	3h vs 0h
82	B4.b8	CD37 molecule	*CD37*	0.0310984	-2.02	3h vs 0h
83	B1.e6	GATA binding protein 4 (GATA4)		0.0216664	-2.04	3h vs 0h
84	B2.e8	hypothetical protein	*LOC510651*	0.0131737	-2.07	3h vs 0h
85	D11.h2	chemokine (C-X-C motif) receptor 3	*CXCR3*	0.0407250	-2.42	3h vs 0h
86	C6.g9	similar to GMCSFR receptor	*LOC511847*	0.0034409	-2.46	3h vs 0h
87	C8.c6	similar to chemokine CCL1/I-309 | similar to chemokine CCL1/I-309	*LOC786156 | LOC786204*	0.0455247	-2.57	3h vs 0h
88	B2.g9	bone morphogenetic protein 4	*BMP4*	0.0380950	-2.85	3h vs 0h

Rank	BOTL-5 coordinates	Feature description	Gene symbol	*P*-value	Fold-change	Time point comparison
1	C9.b5	similar to Interferon-induced guanylate-binding protein 1 (GTP-binding protein 1) (Guanine nucleotide-binding protein 1) (HuGBP-1)	*LOC512486*	0.0320900	3.24	12h vs 3h
2	D1.i2	DNA fragmentation factor, 45kDa, alpha polypeptide	*DFFA*	0.0251619	3.11	12h vs 3h
3	B3.h1	activin A receptor, type I	*ACVR1*	0.0397434	2.99	12h vs 3h
4	C1.g4	similar to Carbonyl reductase 4		0.0445068	2.39	12h vs 3h
5	C10.c5	similar to leukocyte differentiation antigen CD84	*LOC510910*	0.0184758	2.19	12h vs 3h
6	A2.b6	BOTL0100012_A12		0.0172106	2.01	12h vs 3h
7	A5.b6	BOTL0100009_E09		0.0251325	1.85	12h vs 3h
8	A9.b7	CCAAT/enhancer binding protein (C/EBP), beta	*CEBPB*	0.0376696	1.80	12h vs 3h
9	A11.g5	adenylate cyclase 7	*ADCY7*	0.0208110	1.78	12h vs 3h
10	C11.e8	similar to ZNF786 protein	*LOC506408*	0.0085415	1.71	12h vs 3h
11	A5.c4	hypothetical protein	*LOC614103*	0.0456471	1.70	12h vs 3h
12	B11.f7	similar to membrane component chromosome 11 surface marker 1		0.0005171	1.66	12h vs 3h
13	B10.a6	similar to KIAA0691 protein	*LOC527305*	0.0216284	1.66	12h vs 3h
14	A9.a5	BOTL0100002XA10R		0.0476251	1.59	12h vs 3h
15	D12.f3	BOTL0100009_C01		0.0155669	1.57	12h vs 3h
16	B4.g3	similar to Runt domain containing protein	*LOC617389*	0.0189017	1.57	12h vs 3h
17	D11.f1	BOTL0100004XA05R		0.0352874	1.55	12h vs 3h
18	A3.h1	CD34 molecule	*CD34*	0.0453671	1.52	12h vs 3h
19	A12.f1	hypothetical protein	*LOC508039*	0.0174107	1.40	12h vs 3h

Rank	BOTL-5 coordinates	Feature description	Gene symbol	*P*-value	Fold-change	Time point comparison
20	B11.f8	similar to U2 small nuclear RNA auxiliary factor 1-like 2	*LOC535064*	0.0004531	1.37	12h vs 3h
21	B12.a5	hypothetical protein	*LOC523618*	0.0383704	1.37	12h vs 3h
22	A3.e4	monocyte-chemoattractant protein-1 precursor	*CCL2*	0.0359519	1.35	12h vs 3h
23	A4.e1	ELK1, member of ETS oncogene family, mRNA (ELK1		0.0428107	1.34	12h vs 3h
24	D10.b5	BOTL0100009_B10		0.0414188	1.31	12h vs 3h
25	B9.i7	chemokine (C-C motif) ligand 25 (CCL25)		0.0481403	1.27	12h vs 3h
26	C10.a4	similar to Poly [ADP-ribose] polymerase 10 (PARP-10)	*LOC510991*	0.0460924	1.25	12h vs 3h
27	C3.a5	similar to protein phosphatase 1, regulatory subunit 9B	*LOC787141*	0.0479989	-1.22	12h vs 3h
28	D3.f3	similar to human immunity associated protein 1	*LOC615733*	0.0044092	-1.29	12h vs 3h
29	B4.b6	BOTL0100013_E09		0.0298447	-1.37	12h vs 3h
30	D8.f8	BOTL0100009_A11		0.0288457	-1.39	12h vs 3h
31	B1.a7	Transcribed locus, strongly similar to XP_001107198.1 cyclin-dependent kinase inhibitor 2B (p15, inhibits CDK4) isoform 1 [Macaca mulatta]		0.0214954	-1.41	12h vs 3h
32	A6.e8	hypothetical protein	*LOC614141*	0.0181110	-1.43	12h vs 3h
33	A6.a3	hypothetical protein	*LOC614141*	0.0397932	-1.43	12h vs 3h
34	B1.f3	Transcribed locus, strongly similar to XP_001107198.1 cyclin-dependent kinase inhibitor 2B (p15, inhibits CDK4) isoform 1 [Macaca mulatta]		0.0391129	-1.45	12h vs 3h
35	C1.f2	similar to transcriptional regulator protein		0.0451881	-1.45	12h vs 3h
36	A9.e9	hypothetical protein	*LOC514346*	0.0386235	-1.46	12h vs 3h
37	C1.a8	BOTL0100002XD08R		0.0274202	-1.51	12h vs 3h

Rank	BOTL-5 coordinates	Feature description	Gene symbol	*P*-value	Fold-change	Time point comparison
38	B7.a4	Transcribed locus, moderately similar to XP_001113742.1 similar to WD repeat and FYVE domain containing 3, partial [Macaca mulatta]		0.0266146	-1.53	12h vs 3h
39	D5.h9	casein kinase 1, gamma 1	*CSNK1G1*	0.0355179	-1.64	12h vs 3h
40	D5.i4	ribosomal protein L18a	*RPL18A*	0.0239757	-1.65	12h vs 3h
41	B8.e2	granulocyte colony stimulating factor	*CSF3*	0.0256316	-1.68	12h vs 3h
42	B5.i8	hypothetical protein	*LOC515777*	0.0118895	-1.70	12h vs 3h
43	C9.a5	similar to heterogeneous nuclear ribonucleoprotein D-like	*LOC534770*	0.0489859	-1.70	12h vs 3h
44	A4.e9	similar to SFPQ protein	*LOC618226*	0.0339789	-1.72	12h vs 3h
45	A5.f7	ribosomal protein L10	*RPL10*	0.0442826	-1.81	12h vs 3h
46	D3.i4	max-associated protein (MAD4)		0.0129876	-1.84	12h vs 3h
47	D6.d9	similar to neuropilin-1	*LOC539369*	0.0283286	-1.87	12h vs 3h
48	B8.i4	granulocyte colony stimulating factor	*CSF3*	0.0074946	-1.92	12h vs 3h
49	A5.f3	eukaryotic translation elongation factor 1 alpha 1	*EEF1A1*	0.0329128	-2.05	12h vs 3h
50	D9.f1	similar to HS6ST1 protein	*LOC518563*	0.0475204	-2.20	12h vs 3h
51	A5.b5	hypothetical protein	*LOC617051*	0.0441919	-2.21	12h vs 3h
52	B6.d9	actin, beta	*ACTB*	0.0446725	-2.35	12h vs 3h
53	D2.i4	mitogen-activated protein kinase kinase kinase (MAP3K1)		0.0336032	-2.44	12h vs 3h
54	A5.f8	hypothetical protein	*LOC617051*	0.0343348	-2.81	12h vs 3h
55	D8.d3	ADP-ribosyltransferase (NAD+) poly (ADP-ribose) polymerase)	*PARP1*	0.0184160	-2.97	12h vs 3h
56	D8.h5	ADP-ribosyltransferase (NAD+) poly (ADP-ribose) polymerase)	*PARP1*	0.0247990	-3.01	12h vs 3h
